# Asparagine promotes cancer cell proliferation through use as an amino acid exchange factor

**DOI:** 10.1038/ncomms11457

**Published:** 2016-04-29

**Authors:** Abigail S. Krall, Shili Xu, Thomas G. Graeber, Daniel Braas, Heather R. Christofk

**Affiliations:** 1 Department of Molecular and Medical Pharmacology, David Geffen School of Medicine, University of California, 650 Charles East Young Drive South, Los Angeles, California 90095, USA; 2 Crump Institute for Molecular Imaging, David Geffen School of Medicine, University of California, 650 Charles East Young Drive South, Los Angeles, California 90095, USA; 3 Jonsson Comprehensive Cancer Center, David Geffen School of Medicine at UCLA, 650 Charles East Young Drive South, Los Angeles, California 90095, USA; 4 UCLA Metabolomics Center, 650 Charles East Young Drive South, Los Angeles, California 90095, USA; 5 Eli and Edythe Broad Center of Regenerative Medicine and Stem Cell Research, University of California, 650 Charles East Young Drive South, Los Angeles, California 90095, USA

## Abstract

Cellular amino acid uptake is critical for mTOR complex 1 (mTORC1) activation and cell proliferation. However, the regulation of amino acid uptake is not well-understood. Here we describe a role for asparagine as an amino acid exchange factor: intracellular asparagine exchanges with extracellular amino acids. Through asparagine synthetase knockdown and altering of media asparagine concentrations, we show that intracellular asparagine levels regulate uptake of amino acids, especially serine, arginine and histidine. Through its exchange factor role, asparagine regulates mTORC1 activity and protein synthesis. In addition, we show that asparagine regulation of serine uptake influences serine metabolism and nucleotide synthesis, suggesting that asparagine is involved in coordinating protein and nucleotide synthesis. Finally, we show that maintenance of intracellular asparagine levels is critical for cancer cell growth. Collectively, our results indicate that asparagine is an important regulator of cancer cell amino acid homeostasis, anabolic metabolism and proliferation.

## Abstract (web summary)

Cancer cells have been shown to be dependent upon glutamine for growth. Here, the authors show that intracellular asparagine, a glutamine-derived metabolite, is critical to cancer cell growth and can compensate glutamine deficiency by acting as an amino acid exchange factor.

## Graphical abstract

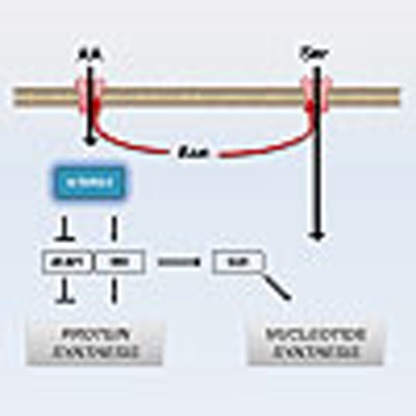

Many tumour cells exhibit high rates of glutamine consumption to support macromolecular biosynthesis and cell proliferation^1^. Glutamine fuels the tricarboxylic acid (TCA) cycle through anaplerosis and contributes to the synthesis of lipids, nucleotides and non-essential amino acids. However, the full spectrum of glutamine contribution to cancer cell growth remains an area of active investigation. Although glutamine can contribute to synthesis of several amino acids through its catabolism to glutamate, only asparagine requires glutamine for *de novo* synthesis; glutamine is a substrate for asparagine synthetase (ASNS). ASNS activity is unidirectional and ATP-dependent, suggesting that cells synthesize asparagine at the expense of macromolecule synthesis and cellular energy.

The importance of asparagine for tumour growth has been demonstrated by the effectiveness of extracellular asparaginase in treating low-ASNS-expressing leukaemia. Notably, the off-target glutaminase (GLS) activity of asparaginase is not required for its anti-tumour effects^2^. Although asparaginase is effective as a therapeutic for cancers that obtain the majority of their asparagine from the environment, cancers that are capable of synthesizing asparagine *de novo* via ASNS are less responsive to asparaginase therapy^3^. Moreover, leukaemic asparaginase resistance is associated with elevated ASNS expression^4^, and ASNS expression in solid tumours correlates with tumour grade and poor prognosis^5^. Recently, genetic silencing of ASNS in sarcoma cells combined with depletion of plasma asparagine levels via asparaginase was shown to blunt tumour growth *in vivo*^6^. Thus, cancer cells appear to have high demand for asparagine, and this demand has the potential to be exploited therapeutically.

The nature of ASNS regulation suggests that asparagine may play a role in cellular amino acid homeostasis. ASNS expression is upregulated in response to individual or combined limitation of numerous amino acids, including most essential amino acids^7^,^8^. Amino acid-starvation-induced upregulation of ASNS is mediated by activating transcription factor 4 (ATF4), the transcriptional activity of which is activated in response to uncharged tRNAs. Although ATF4 regulates expression of genes involved in multiple amino acid synthesis and transport pathways, asparagine alone rescues the impaired proliferation and autophagy resulting from induced ATF4 knockdown^9^, supporting the idea that asparagine globally impacts intracellular amino acid levels. However, the only currently known function for asparagine is as a substrate for protein synthesis. Here we identify a novel role for asparagine as an amino acid exchange factor. We show that asparagine exchanges with extracellular amino acids to regulate mTOR complex 1 (mTORC1) activation, nucleotide synthesis and proliferation. Our results indicate that glutamine contribution to cancer cell survival and proliferation is, in part, mediated by glutamine-dependent asparagine synthesis.

## Results

### Glutamine independence confers asparagine dependence

The broad contribution of glutamine carbon and nitrogen to cancer metabolism has led to the development of drugs targeting glutamine metabolism such as CB-839, an orally bioavailable GLS inhibitor currently undergoing Phase I evaluation for cancer treatment^10^,^11^,^12^. We hypothesized that glutamine-dependent cancers may generate resistance to drugs targeting glutamine metabolism by increasing reliance on metabolites downstream of glutamine. To test this hypothesis, we generated both LPS2 liposarcoma cells that grow in the absence of glutamine and SUM159PT breast cancer cells that are resistant to CB-839 (ref. 10; Supplementary Fig. 1a).

**Figure 1:**
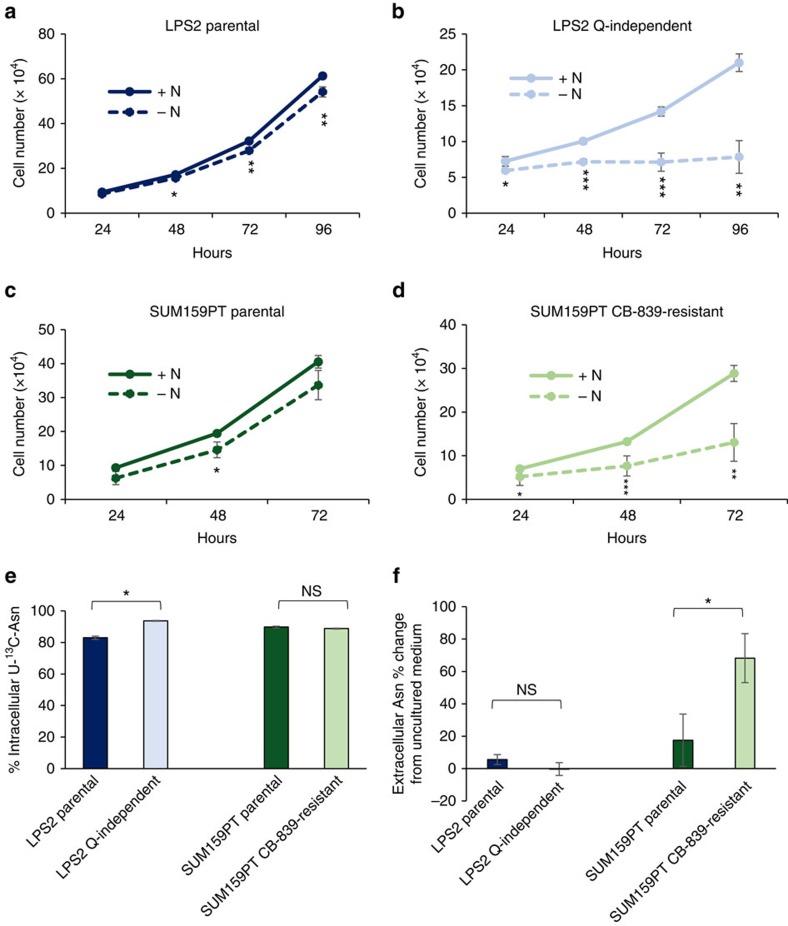
Resistance to glutamine withdrawal or glutaminase inhibition causes cellular asparagine dependence. (**a**–**d**) Proliferation curves of LPS2 parental, LPS2 glutamine (Q)-independent, SUM159PT parental and SUM159PT CB-839-resistant cells in the presence or absence of 0.1 mM asparagine (N) in the medium. (**e**) Percentages of intracellular ^13^C-labelled asparagine in LPS2 parental and glutamine-independent, as well as SUM159PT parental and CB-839-resistant cells labelled with U-^13^C-asparagine in the medium for 24 h, as determined by LC-MS. (**f**) The per cent change in medium asparagine levels as determined by LC-MS after 24-h incubation time for the indicated cells or for medium in an empty tissue culture plate (blank). Error bars denote s.d. of the mean (*n*=3). *P* values were calculated by the Student's t-test: *P<0.05; **P<0.01; ***P<0.001; NS, not significant.

Since glutamine is required for *de novo* asparagine synthesis, we examined whether resistance to glutamine withdrawal confers growth dependence on exogenous asparagine. Also, since CB-839-resistant cells downregulate cellular glutamine consumption (Supplementary Fig. 1b), thereby limiting glutamine availability for the ASNS reaction, we examined whether resistance to GLS inhibition confers growth dependence of exogenous asparagine as well. LPS2 glutamine-independent and SUM159PT CB-839-resistant cells, but not their parental cells, require asparagine in the cell culture medium for proliferation (Fig. 1a–d). LPS2 glutamine-independent cells increase expression of glutamine synthetase (GS) (Supplementary Fig. 1c), likely to fulfil cellular glutamine requirements for nucleotide and protein synthesis by synthesizing glutamine from glutamate^13^. However, the dependence of glutamine-independent cells on exogenous asparagine indicates that GS-derived glutamine is insufficient to fulfil the cellular demand for asparagine and suggests that maintaining intracellular asparagine levels is critical for proliferation. Dependence of glutamine-independent cells on exogenous asparagine for proliferation is consistent with a recent report that exogenous asparagine protects cells from apoptosis on glutamine deprivation^5^.

Given the structural similarity of asparagine and glutamine, we examined whether asparagine could be metabolized like glutamine as a potential resistance mechanism to glutamine deprivation and GLS inhibition. To determine if blocking glutamine metabolism causes cells to catabolize asparagine, we labelled the cell culture medium with either asparagine uniformly labelled on carbon (U-^13^C-asparagine) or uniformly labelled on nitrogen (U-^15^N-asparagine). The only detected metabolites labelled with asparagine carbon in the resistant cells are small percentages of aspartate and malate (Supplementary Fig. 1d). Asparagine nitrogen is detected in ∼10% of purines (Supplementary Fig. 1e). However, both the U-^13^C-asparagine and U-^15^N-asparagine utilized in these experiments are contaminated with small percentages of labelled aspartate, and the observed label on aspartate, malate and purines is likely due to this contamination and the increased consumption of aspartate by the resistant cells (Supplementary Fig. 1f). These data suggest that asparagine is not widely catabolized in LPS2 glutamine-independent cells, SUM159PT CB-839-resistant cells or their parental counterparts.

Since cells resistant to glutamine withdrawal and GLS inhibition require exogenous asparagine for proliferation but do not seem to catabolize it, we considered that the resistant cells may require exogenous asparagine simply for protein synthesis. To examine this possibility, we tested whether the LPS2 cells resistant to glutamine withdrawal and/or the SUM159PT cells resistant to GLS inhibition consume more ^13^C-asparagine from the medium than their parental counterparts. All four cell lines (both resistant cells and both parental cells) obtain a majority of their intracellular asparagine from the culture medium, as indicated by the high percentage of ^13^C-labelled intracellular asparagine when the medium is labelled with U-^13^C-asparagine (Fig. 1e). However, asparagine is not depleted from the medium in either the parental or resistant cell lines, and CB-839-resistant cells actually exhibit a net efflux of asparagine rather than influx when grown in the presence of glutamine (Fig. 1f). These results suggest that dependence of glutamine-independent cells on exogenous asparagine is not solely for the purpose of protein synthesis and suggest that asparagine export may be important for growing cells.

### Asparagine levels impact cancer cell proliferation

**Figure 2:**
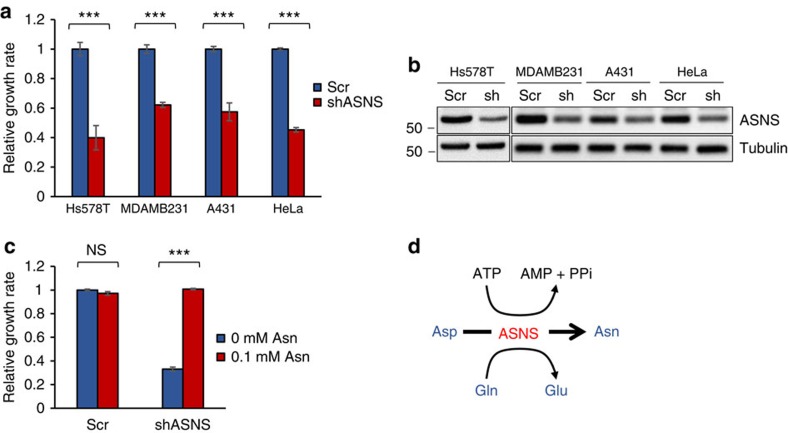
Asparagine levels regulate cell proliferation. (**a**) Relative growth rates of cancer cell lines stably expressing scrambled shRNA (Scr) or ASNS shRNA. Growth rates are normalized to the scrambled shRNA control for each cell line. (**b**) Immunoblot showing ASNS protein levels on stable expression of scrambled (Scr) or ASNS shRNA in cell lines shown in **a**. (**c**) Relative growth rates of HeLa cells stably expressing scrambled shRNA (Scr) or ASNS shRNA in the presence or absence of 0.1 mM asparagine. (**d**) Reaction catalysed by ASNS. Aspartate (Asp) and glutamine (Gln) are converted to asparagine (Asn) and glutamate (Glu). Error bars denote s.d. of the mean (*n*=3). *P* values were calculated by the Student's *t*-test: ***P<0.001; NS, not significant.

Since exogenous asparagine is required for proliferation of glutamine-independent cells, we next asked whether intracellular asparagine levels affect cell proliferation in glutamine-abundant conditions. To address this question, we knocked down ASNS using stable expression of short hairpin RNA (shRNA) in a panel of cancer cell lines. All of the cell lines analysed, when grown in DMEM (which has 4 mM glutamine but lacks asparagine), exhibit reduced proliferation following partial ASNS knockdown when compared with cells expressing a scrambled shRNA (Fig. 2a,b). However, supplementation of DMEM medium with 0.1 mM asparagine completely rescues proliferation of HeLa cells in which ASNS expression is suppressed by ASNS knockdown (Fig. 2c). These results indicate that the growth benefit provided by ASNS expression is due to asparagine synthesis, rather than to another aspect of the ASNS reaction, such as cytoplasmic glutamate production (Fig. 2d), and suggest that intracellular asparagine levels impact cancer cell proliferation in glutamine-abundant conditions.

### Asparagine is an amino acid exchange factor

**Figure 3:**
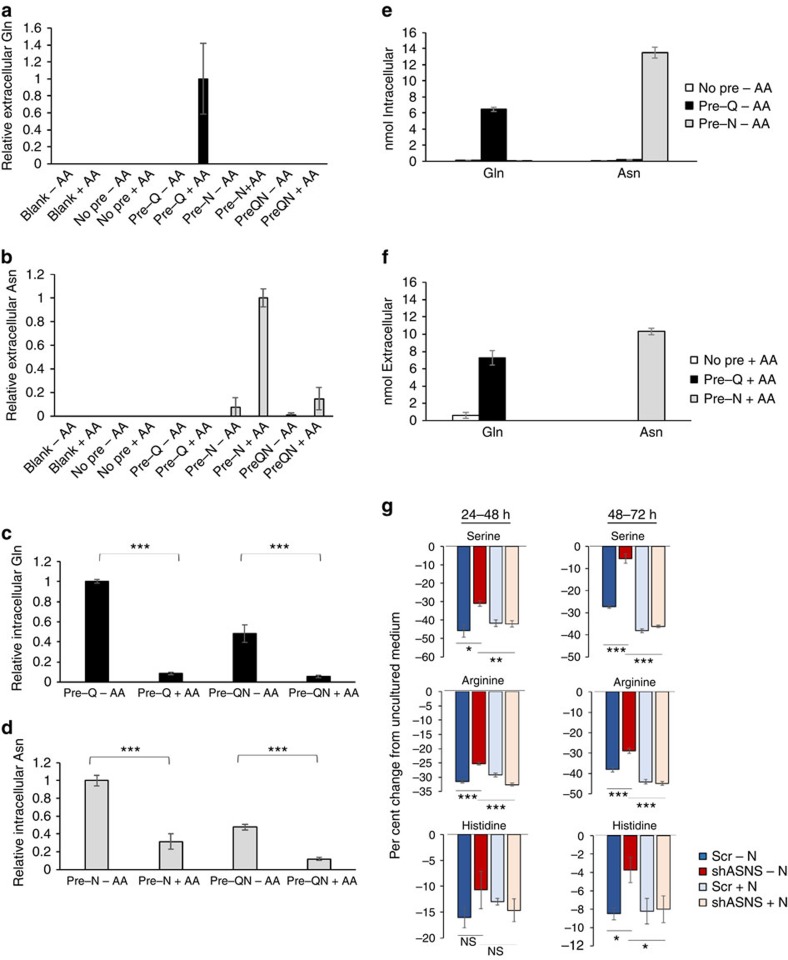
Asparagine is an amino acid exchange factor. Relative glutamine (**a**) and asparagine (**b**) levels in the medium from LPS2 cells, as measured by LC-MS, before and after amino acid (AA) stimulation following pre-loading of the cells with glutamine (Q) and/or asparagine (N). Serum- and amino acid-starved LPS2 cells were pre-loaded with 2 mM glutamine, 2 mM asparagine or 2 mM glutamine and 2 mM asparagine for 60 min prior to stimulation for 30 min with an amino acid mixture (AA medium) lacking glutamine and asparagine. ‘Blank' indicates measurements from plates lacking cells. ‘No Pre' indicates measurements from plates of LPS2 cells not pre-loaded with glutamine or asparagine. Relative intracellular glutamine (**c**) and asparagine (**d**) levels as measured by LC-MS in glutamine- and/or asparagine-pre-loaded cells before and after amino acid stimulation. (**e**) Absolute quantification of intracellular glutamine and asparagine in pre-loaded cells prior to amino acid stimulation. (**f**) Absolute quantification of extracellular glutamine and asparagine following amino acid stimulation. (**g**) Changes in extracellular amino acid levels during a 24-h incubation with HeLa cells. The 24-h incubation began either 24 h (left panels) or 48 h (right panels) post doxycycline-induced expression of a scrambled shRNA (Scr) or ASNS shRNA. Values are shown as per cent change from amino acid measurements from identical medium incubated on plates lacking cells, with negative bars indicating cellular consumption and positive bars indicating production. For **a**–**f**, error bars denote s.d. of the mean (*n*=3). For **g**, error bars denote s.e.m. (*n*=6). *P* values were calculated by the Student's *t*-test: *P<0.05; **P<0.01; ***P<0.001. NS, not significant.

Intracellular glutamine has the capacity to exchange with extracellular essential amino acids^14^ and regulate mTOR activity^14^,^15^,^16^. Given that net asparagine influx is not detected, even for cells that require exogenous asparagine for proliferation, we hypothesized that asparagine is imported into the cell only to be exported in exchange for other amino acids. To test whether intracellular asparagine is capable of exchanging with extracellular amino acids, a high intracellular glutamine/asparagine concentration was generated in serum- and amino acid-starved LPS2 cells by a 60 min pre-load with either 2 mM *L*-glutamine, 2 mM *L*-asparagine or a combination of the two. After washing away the exogenous glutamine and/or asparagine, a glutamine/asparagine-free amino acid mixture (AA medium) was added to the cells for 30 min. Glutamine and asparagine export from the cells was evaluated both by examining extracellular glutamine/asparagine levels following the 30 min AA medium treatment and by measuring intracellular glutamine/asparagine levels before and after AA medium treatment. Confirming previous results^14^, glutamine is detected in the medium only on stimulation with amino acids (Fig. 3a). Consistent with exchange factor capacity, asparagine is also detected in the medium on stimulation with amino acids (Fig. 3b). Moreover, intracellular levels of both glutamine and asparagine are depleted following asparagine/glutamine-free amino acid treatment (Fig. 3c,d), and absolute quantification of intracellular and exported glutamine/asparagine verified that intracellular depletion is due to export (Fig. 3e,f). While asparagine and glutamine are exported following amino acid stimulation of pre-loaded LPS2 cells, efflux of glycine, another neutral amino acid, is not detected (Supplementary Fig. 2a). Notably, when pre-loaded with a combination of glutamine and asparagine, asparagine but not glutamine export is detected (Fig. 3a,b). In addition, while a glutamine pre-load is required to observe glutamine export on amino acid treatment, intracellular asparagine depletion occurs in response to amino acid treatment even with an asparagine-starved, no pre-load state (Supplementary Fig. 2b). These results suggest that LPS2 cells can use asparagine as an amino acid exchange factor and may in fact prefer use of asparagine over glutamine for amino acid exchange.

Knowing that asparagine is capable of exchanging with extracellular amino acids, we next examined whether intracellular asparagine levels regulate amino acid import under normal growth (non-starvation) conditions. Amino acid import is considerably impaired on ASNS knockdown in non-starved HeLa cells grown in DMEM (Supplementary Fig. 2c). Import of serine and basic amino acids arginine and histidine is particularly sensitive to ASNS knockdown in DMEM, and supplementing DMEM with 0.1 mM asparagine completely rescues uptake of these amino acids (Fig. 3g). These results indicate that intracellular asparagine depletion through ASNS knockdown impairs amino acid uptake in cells cultured in complete medium, including 4 mM glutamine, suggesting that amino acid exchange with asparagine occurs under normal growth conditions and is not simply a substitute for glutamine exchange in glutamine-depleted conditions.

### Asparagine regulates serine uptake and metabolism

**Figure 4:**
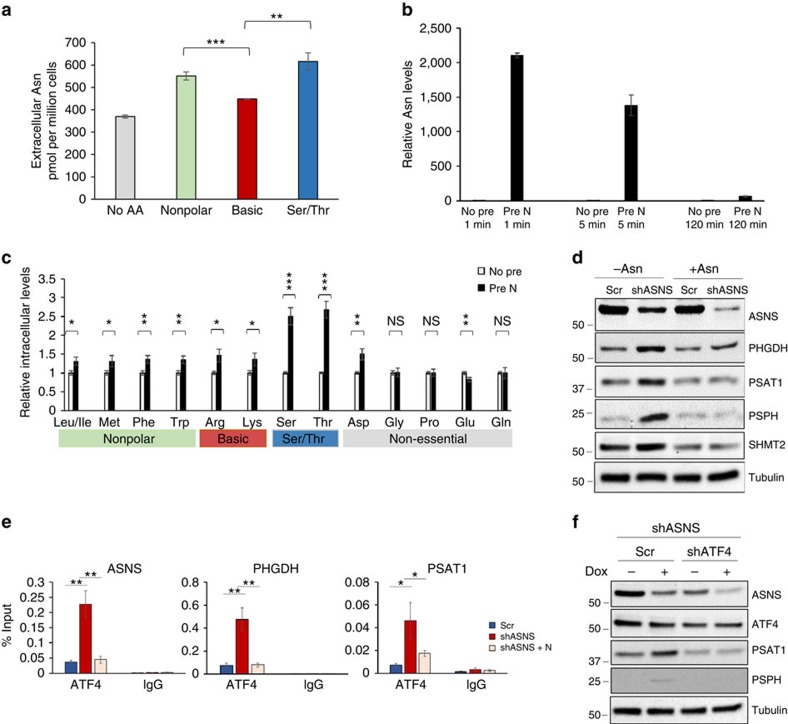
Asparagine levels regulate serine uptake and serine metabolism gene expression. (**a**) Asparagine levels in the media, measured by LC-MS, from serum- and amino acid-starved LPS2 cells pre-loaded with asparagine (Pre N) and unstimulated (No AA) or stimulated with different amino acid sub-categories: non-polar, basic or Ser/Thr. Non-polar amino acids include leucine, isoleucine, methionine, tryptophan and phenylalanine; basic amino acids include lysine, arginine and histidine; Ser/Thr includes serine and threonine. (**b**) Relative intracellular asparagine levels in amino acid-starved glutamine-independent LPS2 cells as measured by LC-MS following 1-, 5- or 120-min stimulation with complete glutamine-free medium. Prior to stimulation, cells were either pre-loaded with asparagine (Pre N) or starve medium (No pre). Declining intracellular asparagine levels over time indicates exchange with extracellular amino acids. (**c**) Relative intracellular amino acid levels in amino acid-starved glutamine-independent LPS2 cells as measured by LC-MS following 5-min stimulation with complete glutamine-free medium. Prior to stimulation, cells were either pre-loaded with asparagine (Pre N) or starve medium (No pre). (**d**) Immunoblot showing serine synthesis pathway protein levels in HeLa cells, 48 h post doxycycline induction of scrambled shRNA or ASNS shRNA expression in the presence or absence of 0.1 mM exogenous asparagine. (**e**) Levels of ATF4 binding to the indicated gene by ChIP–qPCR in HeLa cells 48 h post doxycycline induction of scrambled shRNA or ASNS shRNA expression in the presence or absence of 0.1 mM exogenous asparagine. Chromatin was immunoprecipitated using anti-ATF4 antibody or IgG as a negative control. Values indicate amount of immunoprecipitated DNA as a percentage of input chromatin. (**f**) Immunoblot showing ASNS, ATF4, PSAT1 and PSPH protein levels with or without a 48-h doxycycline induction of ASNS shRNA in HeLa cells stably expressing scrambled or ATF4 shRNA. Error bars denote s.d. of the mean (*n*=3). *P* values were calculated by the Student's *t*-test: *P<0.05; **P<0.01; ***P<0.001. NS, not significant.

Given the capacity of asparagine for amino acid exchange, we asked whether asparagine has increased capacity to exchange with certain amino acids. Notably, ASNS expression in human tumours strongly correlates with expression of genes involved in serine/glycine synthesis and one-carbon metabolism (Supplementary Fig. 3a), suggesting that cellular demand for serine and asparagine may be specifically connected. To examine whether asparagine preferentially exchanges with certain amino acids, asparagine-pre-loaded LPS2 parental cells were treated with non-polar, basic or serine/threonine (which share a transporter) amino acid sub-categories. Although asparagine export is observed with all three groups, asparagine preferentially exchanges with serine/threonine and non-polar amino acids over basic amino acids following amino acid starvation (Fig. 4a). In addition, following a 5-min amino acid stimulation, intracellular non-polar and basic amino acid levels are approximately 50% higher in asparagine-pre-loaded glutamine-independent LPS2 cells compared with cells that lack a pre-load, while intracellular serine and threonine levels are ∼threefold higher in asparagine-pre-loaded cells (Fig. 4b,c).

Because asparagine regulates serine uptake, we asked whether asparagine levels alter serine synthesis or metabolism. To examine this possibility, we determined the impact of ASNS knockdown on serine synthesis pathway gene expression in the absence and presence of exogenous asparagine. ASNS knockdown results in elevated mRNA and protein expression of serine synthesis pathway enzymes, which is prevented by the addition of exogenous asparagine to the medium (Fig. 4d; Supplementary Fig. 3b,c). Consistently, ASNS knockdown results in increased incorporation of glucose carbon into serine and glycine (Supplementary Fig. 3d). ASNS knockdown also results in elevated ATF4 mRNA expression (Supplementary Fig. 3c) and increased localization of ATF4 to the promoters of serine synthesis enzyme genes, as indicated by chromatin immunoprecipitation (Fig. 4e). Moreover, ATF4 knockdown abolishes the increased expression of serine synthesis pathway enzymes on ASNS knockdown (Fig. 4f). These data suggest that cells may compensate for decreased intracellular asparagine levels and consequent decreased ability to exchange intracellular asparagine for extracellular serine by transcriptionally upregulating the serine synthesis pathway through ATF4 activation (Supplementary Fig. 3e).

### Asparagine regulates mTORC1 activation

**Figure 5:**
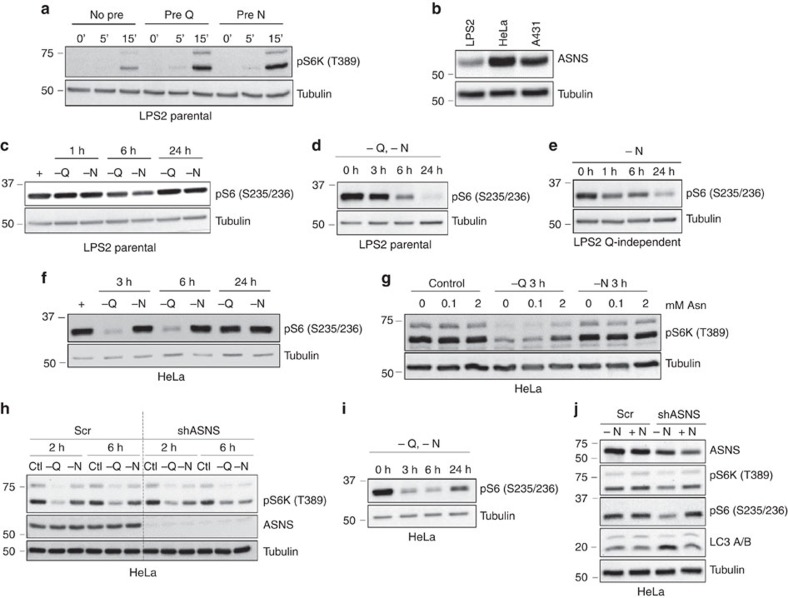
Asparagine regulates mTORC1 activation and autophagy. (**a**) Immunoblotting of lysates from serum- and amino acid-starved LPS2 parental cells pre-loaded with starve medium (No pre), glutamine (Pre Q) or asparagine (Pre N), followed by amino acid stimulation for 0, 5 or 15 min. Lysates were probed with a phospho-specific antibody towards the mTOR target S6K at T389 and with anti-tubulin. (**b**) Immunoblot comparing ASNS levels in lysates from LPS2, HeLa and A431 cells under non-starved conditions. (**c**) Immunoblot showing phosphorylation of downstream mTOR effector S6 ribosomal protein (S235/236) following starvation of LPS2 parental cells of glutamine (−Q) or asparagine (−N) for the indicated times. ‘+' indicates no starvation. (**d**) Immunoblot showing S6 ribosomal protein phosphorylation (S235/236) following glutamine and asparagine starvation of LPS2 parental cells for the indicated times. (**e**) Immunoblot showing S6 ribosomal protein phosphorylation (S235/236) following asparagine starvation of LPS2 glutamine-independent cells for the indicated times. (**f**) Immunoblot showing S6 ribosomal protein phosphorylation (S235/236) following starvation of HeLa cells of glutamine (−Q) or asparagine (−N) for the indicated times. Prior to withdrawal, the medium contained 2 mM glutamine and 0.1 mM asparagine. (**g**) Immunoblot showing S6K phosphorylation (T389) following starvation of HeLa cells of glutamine (−Q) or asparagine (−N) for 3 h. Prior to starvation, cells were cultured for 7 days in DMEM supplemented with the indicated asparagine concentration. (**h**) Immunoblot showing phosphorylation of S6K (T389) in HeLa cells stably expressing scrambled shRNA or ASNS shRNA with and without (Ctl) starvation of glutamine (−Q) or asparagine (−N) for the indicated times. (**i**) Immunoblot showing S6 ribosomal protein phosphorylation (S235/236) following glutamine and asparagine starvation of HeLa cells for the indicated times. (**j**) Immunoblot of HeLa lysates 48 h post doxycycline-induced expression of scrambled shRNA or ASNS shRNA in asparagine-free DMEM (−N) or DMEM supplemented with 0.1 mM asparagine (+N). Lysates were immunoblotted for autophagy marker LC3-II (bottom band), mTOR activity markers pS6K and pS6, and tubulin.

Amino acids are essential for mTORC1 activation^17^,^18^, and mTORC1 activity is particularly sensitive to arginine levels^19^. Because asparagine functions as an amino acid exchange factor and regulates arginine import, we hypothesized that asparagine levels may influence mTORC1 activity. Pre-loading serum- and amino acid-starved LPS2 cells with asparagine prior to stimulation with amino acids increases mTORC1 activation on amino acid stimulation (Fig. 5a; Supplementary Fig. 4a,b), and asparagine and glutamine pre-loading activate mTORC1 with similar kinetics (Fig. 5a). Importantly, asparagine/glutamine pre-load only results in mTOR activation following amino acid stimulation (Fig. 5a), indicating that it is their exchange factor roles that elicit mTORC1 activation.

Given that cells may obtain asparagine either from the environment or by *de novo* synthesis via ASNS activity, we speculated that ASNS expression levels would determine mTORC1 sensitivity to extracellular asparagine levels. Consistent with this hypothesis, mTORC1 activity in LPS2 cells, which have relatively low ASNS levels (Fig. 5b) and obtain asparagine from the medium, is sensitive to 6 h of asparagine withdrawal when cultured in medium with serum and 2 mM glutamine (Fig. 5c). Moreover, the kinetics of mTORC1 activation of serum- and amino acid-starved LPS2 cells is reduced on stimulation with medium lacking asparagine (Supplementary Fig. 4c). While mTORC1 activity recovers after 24 h of asparagine depletion (Fig. 5c), simultaneous withdrawal of both asparagine and glutamine prevents recovery after 24 h of withdrawal (Fig. 5d), suggesting either that asparagine and glutamine can compensate for each other in the exchange factor role or that ASNS activity increases with prolonged asparagine withdrawal. Not surprisingly, the effect of asparagine withdrawal on mTORC1 activity is more pronounced in glutamine-independent (asparagine-dependent) LPS2 cells than in the parental cell line (Fig. 5e versus Fig. 5c and Supplementary Fig. 4d versus Supplementary Fig. 4c). Together these data suggest that mTORC1 activity in cells with low ASNS expression is sensitive to exogenous asparagine levels.

We next evaluated mTORC1 sensitivity to extracellular glutamine and asparagine levels in cells with high ASNS expression and the capacity to synthesize asparagine *de novo*. HeLa and A431 cells, which have relatively high ASNS levels (Fig. 5b), are unaffected by asparagine withdrawal when grown in DMEM supplemented with 0.1 mM asparagine prior to withdrawal (Fig. 5f,g; Supplementary Fig. 4e). Unlike LPS2 mTORC1 activity (Fig. 5c), HeLa and A431 mTORC1 activities are remarkably sensitive to glutamine withdrawal (Fig. 5f,g; Supplementary Fig. 4e). Asparagine (2 mM) supplementation, however, rescues HeLa and A431 mTORC1 activity on glutamine withdrawal (Fig. 5g; Supplementary Fig. 4e). Asparagine rescue of mTORC1 activity in HeLa and A431 cells suggests either that the added asparagine compensates for the exchange factor role of glutamine or that mTORC1 responsiveness to glutamine is mediated, at least partially, by glutamine-dependent asparagine synthesis, which is not required when exogenous asparagine is provided.

To determine if mTORC1 insensitivity to asparagine withdrawal in HeLa cells is due the ability of the cells to synthesize asparagine *de novo*, we reduced ASNS expression and examined mTORC1 sensitivity to exogenous glutamine and exogenous asparagine. Stable ASNS knockdown in high-ASNS-expressing HeLa and A431 cells generates mTORC1 sensitivity to exogenous asparagine and decreases sensitivity to exogenous glutamine (Fig. 5h; Supplementary Fig. 4f). In addition, although HeLa mTORC1 activity recovers after 24 h of glutamine withdrawal (Fig. 5f), simultaneous withdrawal of glutamine and asparagine prevents or delays complete recovery (Fig. 5i), suggesting that mTORC1 recovery is due to increased asparagine consumption in the absence of glutamine. Collectively these results suggest that intracellular asparagine levels can influence mTORC1 activity in cultured cancer cell lines, and the degree to which cellular mTORC1 activity is sensitive to exogenous asparagine depends on the ASNS expression level.

To assess how acute loss of ASNS expression in high-ASNS-expressing HeLa cells affects mTORC1 activation, we examined mTORC1 signalling in HeLa cells with a doxycycline-inducible ASNS shRNA or a doxycycline-inducible scrambled shRNA at early time points post induction of ASNS knockdown. At 48 h post induction of ASNS knockdown in asparagine-free DMEM, ASNS knockdown cells exhibit increased autophagy (as indicated by increased LC3-II levels) and reduced mTORC1 activity (Fig. 5j), consistent with a starved cellular state in the absence of asparagine.

Asparagine levels also influence activation of AMPK, as indicated by increased phosphorylation of AMPKα on Thr172 on induction of ASNS knockdown (Supplementary Fig. 4g,h). Although AMPK activation negatively regulates mTORC1 activity, the timing of AMPK activation is delayed relative to impaired mTORC1 activation on ASNS knockdown (Supplementary Fig. 4g). Moreover, reduced mTORC1 activation is seen to the same extent in ASNS single knockdown and ASNS/AMPKα double knockdown HeLa cells (Supplementary Fig. 4h), suggesting that AMPK activation is not primarily responsible for altered mTORC1 activation with varying intracellular asparagine.

### Asparagine coordinates protein and nucleotide synthesis

**Figure 6:**
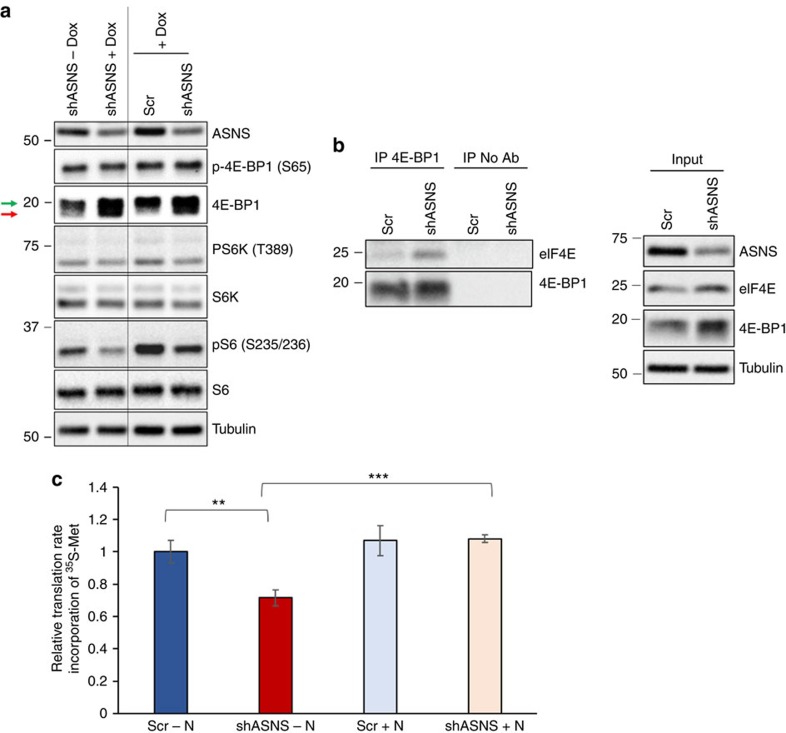
Asparagine levels regulate mRNA translation. (**a**) Immunoblot of HeLa lysates with (shASNS+Dox) and without (shASNS−Dox) a 48-h induction of ASNS shRNA expression and HeLa lysates 48 h after doxycycline induction of scrambled shRNA (Scr) or ASNS shRNA (shASNS). Lysates were immunoblotted for ASNS, phospho-4E-BP1 (S65), total 4E-BP1, phospho-S6K (T389), total S6K, phospho-S6 (S235/236), total S6 and tubulin. Green arrow indicates phosphorylated form of 4E-BP1; red arrow indicates unphosphorylated form. (**b**) Immunoblot showing levels of eIF4E and 4E-BP1 immunoprecipitated with anti-4E-BP1 antibody (left panels) and in the input lysate (right panels) from HeLa cells 48 h after doxycycline induction of scrambled shRNA (Scr) or ASNS shRNA (shASNS). (**c**) Relative rates of ^35^S-methionine incorporation into newly synthesized protein in HeLa cells 72 h after doxycycline induction of scrambled shRNA (Scr) or ASNS shRNA (shASNS). Error bars denote s.d. of the mean (*n*=3). *P* values were calculated by the Student's *t*-test: **P<0.01; ***P<0.001.

To better understand the physiological importance of intracellular asparagine, we examined the influence of asparagine on protein synthesis, a process that is regulated by mTORC1 (ref. 20). Since mTORC1 promotes mRNA translation through phosphorylation of eukaryotic initiation factor 4E (eIF4E)-binding protein (4E-BP1), with phosphorylation preventing 4E-BP1 from binding to eIF4E at the 5′ cap of mRNAs and permitting assembly of the translation initiation complex^21^, we examined whether ASNS knockdown impacts 4E-BP1 phosphorylation. Although absolute levels of 4E-BP1 phosphorylated on Ser65 are unaltered, ASNS knockdown leads to elevated levels of unphosphorylated 4E-BP1, resulting in a decreased ratio of phosphorylated-to-unphosphorylated 4E-BP1 (Fig. 6a) and increased 4E-BP1-eIF4E binding, as indicated by co-immunoprecipitation (Fig. 6b). The gene encoding 4E-BP1 is an ATF4 target^22^,^23^, and elevated levels of unphosphorylated 4E-BP1 may be a result of ATF4 activation induced by asparagine depletion (Fig. 3e). Consistent with an increased percentage of the inhibitory form of 4E-BP1, ASNS knockdown reduces the rate of ^35^S-methionine incorporation into newly synthesized proteins, in a manner that is completely rescued by supplementing the medium with 0.1 mM asparagine (Fig. 6c). Together, these results are consistent with a model whereby intracellular asparagine levels regulate protein synthesis through modulation of mTORC1 phosphorylation of 4E-BP1.

**Figure 7:**
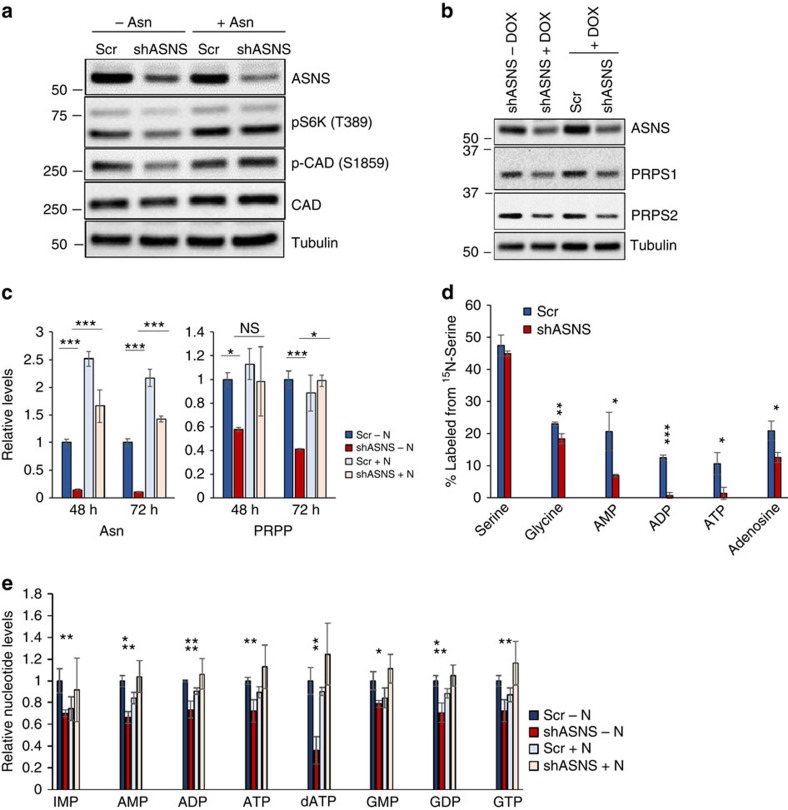
Asparagine levels regulate nucleotide synthesis. (**a**) Immunoblot of HeLa lysates 48 h post doxycycline-induced expression of scrambled shRNA or ASNS shRNA in asparagine-free DMEM (−Asn) or DMEM supplemented with 0.1 mM asparagine (+Asn). Lysates were immunoblotted for mTOR activity marker pS6K, phospho-CAD (S1859), total CAD and tubulin. (**b**) Immunoblot of HeLa lysates with (shASNS+Dox) and without (shASNS−Dox) a 48-h induction of ASNS shRNA expression and HeLa lysates 48 h after doxycycline induction of scrambled shRNA (Scr) or ASNS shRNA (shASNS). Lysates were immunoblotted for ASNS, PRPS1, PRPS2 and tubulin. (**c**) Relative levels of asparagine and PRPP extracted from HeLa cells 48 and 72 h after doxycycline induction of scrambled shRNA (Scr) or ASNS shRNA expression as measured by LC-MS. (**d**) Percentages of the indicated metabolites labelled with 15 N extracted from HeLa cells labelled with exogenous 15 N-serine (50:50 15 N:14 N) for 24 h at 24 h post induction of scrambled shRNA (Scr) or ASNS shRNA expression. (**e**) Relative levels of the indicated intracellular metabolites extracted from HeLa cells 72 h after doxycycline induction of scrambled shRNA (Scr) or ASNS shRNA expression as measured by LC-MS. Error bars denote s.d. of the mean (*n*=3). *P* values were calculated by the Student's *t*-test: *P<0.05; **P<0.01; ***P<0.001. NS, not significant.

**Figure 8:**
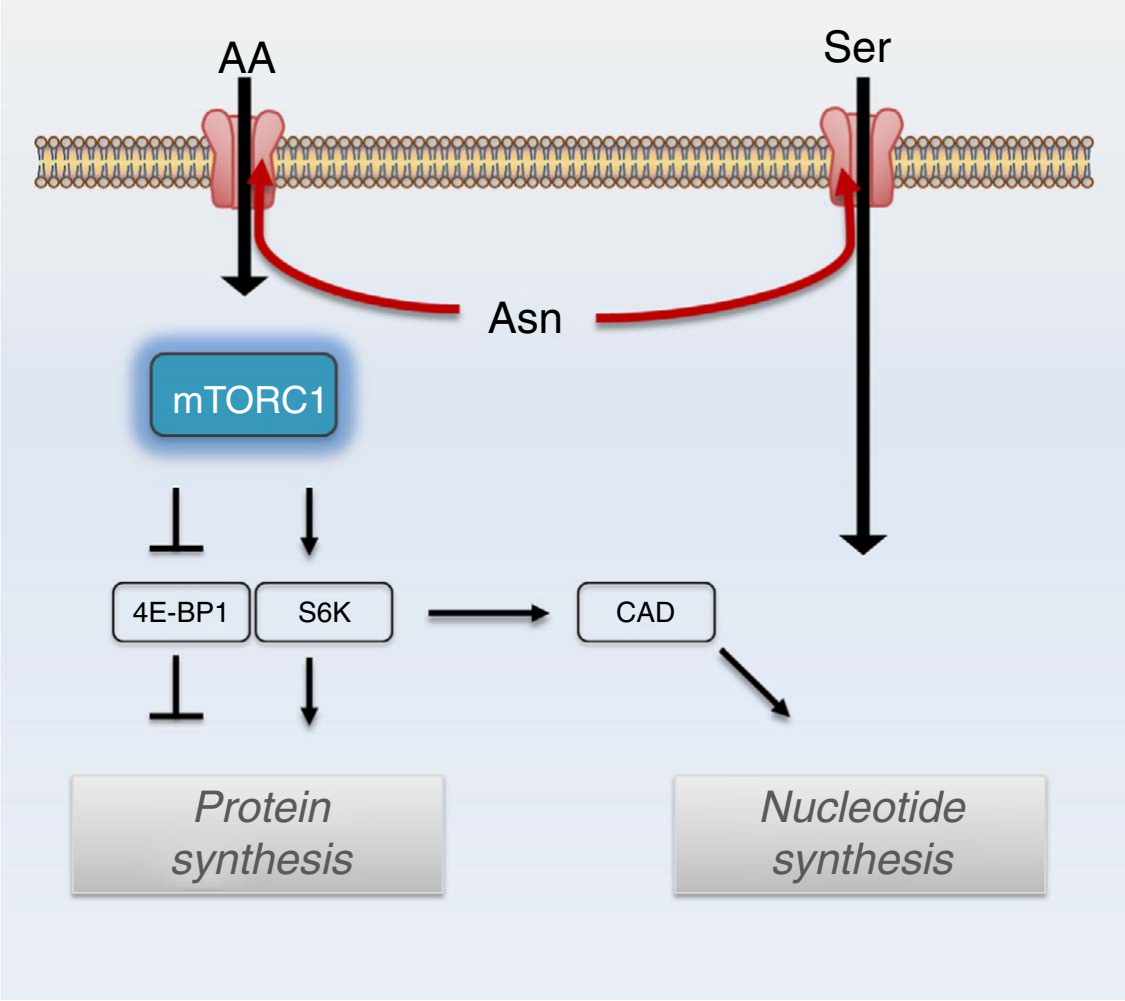
Asparagine coordinates protein and nucleotide synthesis. Model illustrating asparagine contribution to protein synthesis and nucleotide synthesis. Intracellular asparagine exchanges with extracellular amino acids (AA), including serine (Ser), to coordinate protein and nucleotide synthesis.

Because asparagine influences both serine uptake and mTORC1 activity, we examined whether asparagine also affects nucleotide synthesis. Serine is crucial for both purine and thymidine synthesis through its conversion to glycine and donation to the one-carbon unit pool^24^. mTORC1 activation promotes nucleotide synthesis by increasing phosphorylation of CAD (carbamoyl-phosphate synthetase 2, aspartate transcarbamoylase, dihydroorotase)^25^, which catalyses the first three steps of *de novo* pyrimidine synthesis, and by increasing translation of phosphoribosyl pyrophosphate (PRPP) synthetase 2 (PRPS2)^26^, which synthesizes PRPP, a substrate for the rate limiting enzymes of purine and pyrimidine synthesis, as well as for nucleotide salvage pathway enzymes. Reducing intracellular asparagine through ASNS knockdown results in reduced phosphorylation of CAD on serine 1859 and reduced PRPS2 protein levels (Fig. 7a,b). PRPS1 protein levels (Fig. 7b) and PRPS1 and PRPS2 mRNA levels (Supplementary Fig. 5a), however, are also reduced on ASNS knockdown, suggesting that mTORC1 translational regulation is not entirely responsible for decreased PRPS levels. Consistent with the observed reduction in CAD phosphorylation, PRPS1/2 levels and serine uptake in ASNS knockdown cells, ASNS knockdown results in reduced PRPP levels (Fig. 7c), decreased serine incorporation into purines (Fig. 7d), reduced purine levels (Fig. 7e) and reduced pyrimidine levels (Supplementary Fig. 5b). Although both the reduction in serine import and reduced PRPS levels may contribute to reduced nucleotide levels on ASNS knockdown, rescuing PRPS2 levels through ectopic PRPS2 overexpression is insufficient to rescue nucleotide levels (Supplementary Fig. 5c,d). Rescuing serine uptake with cell-permeable serine methyl-ester, on the other hand, rescues some of the nucleoside monophosphate and nucleoside diphosphate levels, but does not rescue levels of nucleoside triphosphates (Supplementary Fig. 5e). Through its involvement in both mTORC1 activation and serine uptake, our results collectively suggest that asparagine plays a role in coordinating protein and nucleotide synthesis (Fig. 8).

## Discussion

We present here a previously unrecognized role for asparagine as an amino acid exchange factor. Our results suggest that intracellular asparagine exchanges with extracellular amino acids, especially serine, arginine and histidine, to promote mTORC1 activation, protein and nucleotide synthesis and cell proliferation under normal growth non-starvation conditions.

Our results suggest that asparagine is an important glutamine-derived metabolite for proliferating cells, and demand for asparagine likely contributes to cancer cell glutamine dependence, especially under cell culture conditions (which sometimes lack asparagine). Cancer cells that have adapted to glutamine independence require exogenous asparagine for proliferation. Importantly, resistance to glutamine depletion does not occur when cells are cultured in media that lack asparagine, such as DMEM. Although glutamine-independent cells presumably synthesize enough glutamine for protein and purine synthesis via GS, the cells are not able to grow in the absence of exogenous asparagine, indicating that the demand for asparagine in proliferating cells exceeds the amount of asparagine that can be synthesized through the GS/ASNS pathway.

Although both asparagine and glutamine are capable of exchanging with amino acids, the extent to which each amino acid acts as an exchange factor under physiological conditions is unclear. Glutamine is the most abundant free amino acid in the blood at a concentration of 0.5–0.8 mM (ref. 27), and most cell culture media contain 2–4 mM glutamine. Blood asparagine concentration is much lower at 0.05–0.1 mM (ref. 27), and media asparagine concentration ranges from 0 mM in DMEM to 0.38 mM in RPMI. Moreover, we found that intracellular glutamine levels are 10–100-fold higher than asparagine levels, depending on the cell line and the asparagine concentration in the media. However, although glutamine is more abundant than asparagine, glutamine is extensively metabolized—primarily in the mitochondria by GLS. On the other hand, asparagine is a metabolic dead-end and may therefore be more available for exchange with extracellular amino acids. We show that asparagine is preferentially exported on amino acid stimulation following pre-loading of cells with an equimolar asparagine/glutamine combination, and that asparagine, unlike glutamine, is exported even without a pre-load. We also show that reducing intracellular asparagine levels via ASNS knockdown decreases amino acid uptake, even in the presence of abundant glutamine. The impact of ASNS expression levels on mTORC1 activation suggests that the influence of glutamine on mTORC1 activity may be in part mediated by glutamine-dependent asparagine synthesis via ASNS.

Although our data suggests asparagine exchanges with extracellular amino acids, it is unclear which transporters are involved in asparagine import and exchange. Glutamine is thought to be imported into the cell through SLC1A5 (or ASCT2) and exchange with extracellular amino acids through the neutral amino acid antiporter SLC7A5 (or LAT1)^14^. The structural similarity between glutamine and asparagine suggests that the two amino acids may have similar affinities for transporters. In addition, pre-loading cells with glutamine or asparagine results in similar intracellular levels of glutamine or asparagine prior to AA medium stimulation (Fig. 3e); however, pre-loading with both glutamine and asparagine decreases the total intracellular amount of each by about half (Fig. 3c,d), suggesting potential use of a common transporter for import into the cell. Asparagine exchange with serine and arginine raises the possibility that asparagine uses SLC1A4 and SLC7A1 for its exchange function. Future studies assessing asparagine export on inhibition of individual amino acid antiporters will further clarify the mechanistic details of asparagine exchange.

Our data suggest that asparagine plays a role in coordinating protein and nucleotide synthesis (Fig. 8a). The exchange factor role of asparagine influences protein synthesis through amino acid-induced mTORC1 activation and downstream activation of translation initiation factors, such as eIF4E. Asparagine regulation of nucleotide synthesis is likely due to asparagine exchange with extracellular serine. However, our data suggest that asparagine may also influence nucleotide synthesis through modulation of PRPS1/2 levels and/or mTORC1-regulated CAD activity.

Our results support asparagine as an important contributor to cancer cell growth and suggest strategies for improving efficacy of asparaginase and GLS inhibitors as cancer treatments. Our findings that asparagine is an amino acid exchange factor that modulates protein and nucleotide biosynthesis can explain the clinical efficacy of asparaginase in low-ASNS-expressing cancers^3^. Since cancers evade asparaginase sensitivity by upregulating ASNS expression^4^, presumably to recover intracellular asparagine pools via ASNS-catalysed synthesis from glutamine, coupling asparaginase treatment with a glutamine-low diet may improve efficacy of asparaginase treatment. In addition, our findings that cancer cells acquire resistance to CB-839-inhibition by becoming auxotrophic for asparagine suggest that coupling GLS inhibition with an asparagine-low diet may improve efficacy of GLS inhibitors. Collectively, our results suggest that future studies combining drugs targeting asparagine or glutamine metabolism with specialized diets to prevent or delay drug resistance may improve treatment outcomes.

## Methods

### Cell lines and culture conditions

HeLa cells (ATCC) and A431 (provided by Dr Thomas Graeber, UCLA) were cultured in DMEM supplemented with 10% foetal bovine serum and 1% penicillin-streptomycin, unless otherwise stated. The LPS2 cell line was derived from a liposarcoma tumour sample^28^,^29^. SUM159PT (provided by Dr Frank McCormick, UCSF) and LPS2 cells were cultured as described below. Mycoplasma contamination testing was not conducted on cell lines used in this study.

### Generation of glutamine-independence and CB-839 resistance

To generate glutamine-independence, LPS2 liposarcoma cells were cultured in a modified DMEM (m-DMEM) containing or lacking glutamine, with media replacement every 48 h. m-DMEM is glucose/glutamine/pyruvate-free DMEM (Invitrogen A14430-01) supplemented with 10 mM glucose, 1 mM pyruvate, 0.1 mM alanine, 0.1 mM aspartic acid, 0.1 mM asparagine, 0.1 mM glutamic acid, 2 mM glutamine, 0.3 mM proline, 0.00289, mM thymidine and 10% dialyzed FBS (Invitrogen). Renewed proliferation in the absence of glutamine was observed ∼6 weeks post-glutamine withdrawal. To generate resistance to GLS inhibitor CB-839 (Calithera Biosciences), SUM159PT breast cancer cells were cultured in pyruvate-free m-DMEM in the presence of 1 uM CB-839 or an equal volume of DMSO, with media replacement every 48 h. A 10 mM stock solution of CB-839 was prepared in DMSO, and a fresh aliquot was thawed for each media change. Renewed proliferation in the presence of CB-839 was observed ∼6 weeks after treatment was initiated.

### Proliferation assays

Cells were seeded in triplicate in six-well plates at 5 × 10^4^ cells per well. Cells were counted every 24 h for 72 or 96 h using a particle counter (Beckman Coulter). For asparagine withdrawal experiments, cells were seeded in complete medium, allowed to adhere to the plate for ∼6 h, followed by replacement with medium containing or lacking asparagine.

### Intracellular metabolite extraction and analysis

Cells were seeded in six-well plates, and metabolites were extracted at 70–80% confluence. When heavy isotope labelling was performed, medium was replaced 24 h prior to extraction with medium containing the labelled metabolite. Cells were washed with ice-cold 150 mM ammonium acetate, and scraped off the plate in 800 μl ice-cold 50% methanol. About 10 nmol norvaline was added as an internal standard, followed by 400 μl chloroform. After vigorous vortexing, the samples were centrifuged at maximum speed, the aqueous layer was transferred to a glass vial and the metabolites were dried under vacuum. Metabolites were resuspended in 50 μl 70% acetonitrile (ACN) and 5 μl of this solution used for the mass spectrometer-based analysis. The analysis was performed on a Q Exactive (Thermo Scientific) in polarity-switching mode with positive voltage 4.0 kV and negative voltage 4.0 kV. The mass spectrometer was coupled to an UltiMate 3000RSLC (Thermo Scientific) UHPLC system. Mobile phase A was 5 mM NH4AcO, pH 9.9, B was ACN and the separation achieved on a Luna 3 mm NH2 100 A (150 × 2.0 mm) (Phenomenex) column. The flow was 200 μl min^−1^, and the gradient ran from 15% A to 95% A in 18 min, followed by an isocratic step for 9 min and re-equilibration for 7 min. Metabolites were detected and quantified as area under the curve based on retention time and accurate mass (≤3 p.p.m.) using the TraceFinder 3.1 (Thermo Scientific) software. Relative amounts of metabolites between various conditions, as well as percentage of labelling, were calculated and corrected for naturally occurring 13C abundance^30^.

### Medium metabolite measurements

For medium metabolite measurements, 5 μl medium was mixed with 800 μl ice-cold 50% methanol. About 10 nmol norvaline was added as an internal standard, followed by 400 μl chloroform. After vigorous vortexing, the samples were centrifiuged at maximum speed, the aqueous layer was transferred to a glass vial and the metabolites were dried under vacuum. Metabolites were resuspended in 100 μl 70% ACN and 5 μl of this solution used for the mass spectrometer-based analysis, as described above for intracellular metabolites. To look at changes in extracellular amino acid levels over time, the experiment was initiated by the addition of fresh medium to the cells, and blank medium from a cell-free plate was included in the analysis. Data was plotted as per cent change from blank medium and normalized by area under the growth curve during the incubation period. To highlight differences in amino acid uptake, experiments in Fig. 3g and Supplementary Fig. 2c were performed with 1/3 × Amino Acid DMEM (DMEM diluted threefold with 1.8 mM calcium chloride, 0.81 mM magnesium sulfate, 5.33 mM potassium chloride, 110 mM sodium chloride, 0.906 mM sodium phosphate monobasic, 44 mM sodium bicarbonate, 25 mM glucose, 4 mM glutamine and 1 mM pyruvate). For asparagine and glutamine absolute quantification, serial dilutions of U-^13^C-asparagine or U-^13^C-glutamine at known concentrations were added to sample triplicates prior to chloroform addition. Sample asparagine and glutamine amounts were calculated according to standard curves generated from the ^13^C standards. Glutamine consumption and glutamate production rates were determined using a Nova Biomedical BioProfile Basic Analyzer and normalized to area under the growth curve.

### Amino acid starvation and stimulation

Amino acid starvation was performed as previously described^14^. Cells were starved of serum for 16 h followed by a 3 h amino acid starvation in D-PBS containing 0.9 mM calcium chloride, 0.5 mM magnesium chloride, 1 g l^−1^ glucose and 20 mM HEPES pH 7.4 (starve medium). For experiments involving glutamine and asparagine pre-loading, serum- and amino acid-starved cells were incubated with 2 mM glutamine, 2 mM asparagine or 2 mM asparagine and 2 mM glutamine in starve medium for 1 h at 37 °C. Pre-loaded cells were washed twice with PBS to remove residual extracellular glutamine and asparagine prior to amino acid treatment. Amino acid stimulation was performed with AA medium (20 mM HEPES pH 7.4, 1.8 mM calcium chloride, 0.814 mM magnesium sulfate, 5.33 mM KCl, 110.3 mM NaCl, 0.906 mM sodium phosphate monobasic, 44.05 mM sodium bicarbonate, 1 g l^−1^ glucose, 0.8 mM leucine, 0.8 mM isoleucine, 0.2 mM methionine, 0.8 mM valine, 0.4 mM phenylalanine, 0.08 mM tryptophan, 02 mM histidine, and 0.8 mM lysine, 0.4 mM arginine, 0.4 mM serine, 0.8 mM threonine) or glucose/glutamine/pyruvate-free DMEM supplemented with 1 g l^−1^ glucose.

### Cell lysis and immunoblotting

Cells were lysed in buffer containing 50 mM Tris pH 7.4, 1% Nonidet P-40, 0.25% sodium deoxycholate, 1 mM EDTA, 150 mM NaCl, 1 mM dithiothreitol, 1 mM sodium orthovanadate, 20 mM sodium fluoride, 2 μg ml^−1^ aprotinin, 2 μg ml^−1^ leupeptin and 0.7 μg ml^−1^ pepstatin. Western blot analysis was performed using standard protocols, and the following commercial antibodies were used as probes: ASNS (Proteintech 14681-1-AP, 1:1,000), phospho-T389 S6 kinase (Cell Signaling 9234, 1:500), S6 kinase (Cell Signaling 2708, 1:1,000), phospho-S235/235 S6 ribosomal protein (Cell Signaling 4858, 1:3,000), S6 ribosomal protein (Cell Signaling 2217, 1:1,000), LC3A/B (Cell Signaling 4108, 1:1,000), PHGDH (Cell Signaling 13428, 1:1,000), PSAT1 (Abnova H00029968-A01, 1:500), PSPH (Sigma HPA020376, 1:500), SHMT1 (Abcam ab55736, 1:1,000), SHMT2 (Cell Signaling 12762, 1:1,000), PRPS2 (Abnova H00005634-A01, 1:1,000), 4E-BP1 (Cell Signaling 9452, 1:1,000) and α-tubulin (Sigma T6074, 1:10,000). Immunoblot images have been cropped for presentation. Full-size images are presented in Supplementary Fig. 6.

### Quantitative real-time PCR

RNA was purified with Qiagen RNeasy Kit. About 1 μg of total RNA was used to synthesize complementary DNA (cDNA) using the iScript cDNA Synthesis Kit (Bio-Rad) as per manufacturer's instructions. Quantitative PCR (qPCR) was conducted on the Roche LightCycler 480 using SYBR Green I Master Mix (Roche) and 0.5 μM primers. Relative expression values are normalized to control gene (60S acidic ribosomal protein P0). qPCR was performed with the following primers:

ASNS Fwd: 5′-CAGAAGATGGATTTTTGGCTG-3′

ASNS Rev: 5′-TGTCCAGGAAGAAAAGGCTC-3′

PHGDH Fwd: 5′-GCAAAGAGGAGCTGATAGCG-3′

PHGDH Rev: 5′-TTCTCAGCTGCGTTGATGAC-3′

PSAT1 Fwd: 5′-TGCCGCACTCAGTGTTGTTAG-3′

PSAT1 Rev: 5′-GCAATTCCCGCACAAGATTCT-3′

PSPH Fwd: 5′-GAGGACGCGGTGTCAGAAAT-3′

PSPH Rev: 5′-GGTTGCTCTGCTATGAGTCTCT-3′

SHMT1 Fwd: 5′-CTGGCACAACCCCTCAAAGA-3′

SHMT1 Rev: 5′-AGGCAATCAGCTCCAATCCAA-3′

SHMT2 Fwd: 5′-CCCTTCTGCAACCTCACGAC-3′

SHMT2 Rev: 5′-TGAGCTTATAGGGCATAGACTCG-3′

MTHFD2 Fwd: 5′-CTGCGACTTCTCTAATGTCTGC-3′

MTHFD2 Rev: 5′-CTCGCCAACCAGGATCACA-3′

ATF4 Fwd: 5′-GTCCCTCCAACAACAGCAAG-3′

ATF4 Rev: 5′-CTATACCCAACAGGGCATCC-3′

PRPS1 Fwd: 5′-CCTGCTATTTCTCGCATCAA-3′

PRPS1 Rev: 5′-GTGAGTTCTCCTGATGGCTT-3′

PRPS2 Fwd: 5′-CTGGGGCGGATCACATCATC-3′

PRPS2 Rev: 5′-CCGCATACAAATTATCCACAGGA-3′

### Translation rate

Labelling medium was prepared by adding 20 uCi ml^−1 35^S-methionine (PerkinElmer) to methionine-free DMEM containing 10% dialyzed FBS. Following two PBS washes, cells were labelled for 10 min and immediately lysed. About 10 μg of lysate was spotted onto Whatman filters (pre-blocked with 0.1% methionine). Filters were added to ice-cold 10% TCA for 20 min, boiled in 5% TCA for 15 min and washed in ice-cold 5% TCA and 95% ethanol for 20 min each. Scintillation fluid was added to dried filters and radioactivity was measured. Counts per minute readings were normalized to protein content (Bradford assay).

### Chromatin immunoprecipitation and quantitative real-time PCR

About 2 × 10^7^ cells were crosslinked by the addition of 1% formaldehyde-containing medium for 10 min, followed by the addition of 140 mM glycine for 5 min. Crosslinked cells were lysed in 1% SDS; 50 mM Tris-HCl, pH 8; 20 mM EDTA and sonicated to produce DNA fragments between 200 and 600 bp in length. Lysate corresponding to 5 × 10^6^ cells was immunoprecipitated with anti-ATF4 antibody (Proteintech 10835-1-AP) or normal rabbit IgG (Santa Cruz) as a negative control. Complexes were washed with Wash Buffer A (50 mM HEPES, pH 7.9; 0.1% SDS; 1% Triton X-100; 0.1% sodium deoxycholate; 1 mM EDTA; 140 mM NaCl), Wash Buffer B (50 mM HEPES, pH 7.9; 0.1% SDS; 1% Triton X-100; 0.1% sodium deoxycholate; 1 mM EDTA; 500 mM NaCl), LiCl Buffer (20 mM Tris-HCl, pH 8; 0.5% NP-40; 0.5% sodium deoxycholate; 1 mM EDTA; 250 mM LiCl) and TE Buffer (10 mM Tris-HCl, pH 8; 1 mM EDTA), and eluted in Elution Buffer (50 mM Tris-HCl, pH 8; 1 mM EDTA; 1% SDS) followed by TE containing 0.67% SDS for 10 min each at 65 °C. Crosslinking was reversed overnight at 65 °C and samples were treated with RNase A for 60 min and Proteinase K for 90 min. DNA was purified using the QIAGEN PCR Purification Kit. About 2 μl of eluted immunoprecipitated DNA and 2 μl of eluted 1% input were used for Quantitative Real-Time PCR with the following primers sequences:

ASNS Fwd: 5′-TGGTTGGTCCTCGCAGGCAT-3′

ASNS Rev: 5′-CGCTTATACCGACCTGGCTCCT-3′

PHGDH Fwd: 5′-CGTAAGGCAGCAAACACGTA-3′

PHGDH Rev: 5′-CCAGCGATAAACCAAAGGTG-3′

PSAT1 Fwd: 5′-GTTTGCATCCCTGCGTGT-3′

PSAT1 Rev: 5′-CCGAGCTTCCTCACCAACT-3′

### shRNA-mediated knockdown

For stable ASNS knockdown, ASNS shRNA (5′-CCGGGCTGTATGTTCAGAAGCTAAACTCGAGTTTAGCTTCTGAACATACAGCTTTTTG-3′) or a non-specific ‘scrambled' shRNA sequence (5′-CCGGCAACAAGATGAAGAGCACCAACTCGAGTTGGTGCTCTTCATCTTGTTGTTTTT-3′) in the pLKO.1-puro vector (Sigma) were cotransfected in HEK293T cells with expression vectors containing the *gag*/*pol*, *rev* and *vsvg* genes. Lentivirus was harvested 48 h after transfection and added to subconfluent HeLa, A431, Hs578T, MDAMB231 and HCC70 cells with 4 μg ml^−1^ polybrene for 12–16 h. Cells were selected in 2 μg ml^−1^ puromycin for 1 week. For stable ATF4 or AMPKα knockdown, ATF4 shRNA (5′-CCGGGCCAAGCACTTCAAACCTCATCTCGAGATGAGGTTTGAAGTGCTTGGCTTTT-3′), AMPKα shRNA (5′-CCGGTGATTGATGATGAAGCCTTAACTCGAGTTAAGGCTTCATCATCAATCATTTTT-3′) or a non-specific shRNA sequence (5′-CCGGGCGCGATAGCGCTAATAATTTCTCGAGAAATTATTAGCGCTATCGCGCTTTTT-3′) in the pLKO.1-hygro were transfected into HEK293 T as above for lentivirus production. Infected HeLa shASNS cells were selected in 150 μg ml^−1^ Hygromycin B for 2 weeks.

For inducible ASNS knockdown, shRNA oligonucleotides (shASNS 5′-CACCGCTGTATGTTCAGAAGCTAAATTCAAGAGATTTAGCTTCTGAACATACAGC-3′ and 5′-AAAAGCTGTATGTTCAGAAGCTAAATCTCTTGAATTTAGCTTCTGAACATACAGC-3′; and shScramble 5′-CACCGTAGCGACTAAACACATCAATTCAAGAGATTGATGTGTTTAGTCGCTA-3′ and 5′-AAAATAGCGACTAAACACATCAATCTCTTGAATTGATGTGTTTAGTCGCTAC-3′) were annealed and ligated into pENTR/H1/TO vector (Invitrogen #K4920-00) following the BLOCK-iT Inducible H1 RNAi Entry Vector Kit manual. Resulting shRNA constructs were recombined into pLentipuro/BLOCK-iT-DEST using Gateway LR Clonase II (Invitrogen #11791-020). pLentipuro/BLOCK-iT-DEST is a modification of pLenti4/BLOCK-iT-DEST (Invitrogen #K4925-00) wherein the SV40 promoter/zeocin resistance cassette was replaced with the human PGK promoter/puromycin resistance gene and the cPPT/WPRE elements were added, and was kindly provided by Dr Andrew Aplin (Thomas Jefferson University, Kimmel Cancer Center)^31^,^32^. Recombinant lentiviruses were packaged in 293T cells by co-transfecting 4 μg each of lentivirus plasmid with expression vectors containing the *gag*/*pol*, *rev* and *vsvg* genes. Lentivirus was harvested 48 h after transfection and added to subconfluent HeLa cells with 4 μg ml^−1^ polybrene for 16 h. Cells were selected in 2 μg ml^−1^ puromycin for 1 week. Doxycycline induction of knockdown is controlled by the Tet repressor (TetR) protein expressed from the pLenti0.3/EF/GW/IVS-Kozak-TetR-P2A-Bsd vector, which was constructed by Dr Ethan Abel and was kindly provided by Dr Diane M. Simeone (the University of Michigan, Translational Oncology Program). Knockdown was induced with 25 ng ml^−1^ doxycycline.

### ASNS correlation data

ASNS correlations data (Supplementary Fig. 3a) were based on TCGA mRNA expression data via cBioPortal (http://www.cbioportal.org/).

### Data availability

Data referenced in this study are available from the cBioPortal database http://www.cbioportal.org/.

## Additional information

**How to cite this article:** Krall, A. S. *et al.* Asparagine promotes cancer cell proliferation through use as an amino acid exchange factor. *Nat. Commun.* 7:11457 doi: 10.1038/ncomms11457 (2016).

## Supplementary Material

Supplementary Information Supplementary Figures 1-6
