# Supplementary material for: Asparagine promotes cancer cell proliferation through use as an amino acid exchange factor
Source: Nat Commun. 2016 Apr 29;7:11457. doi: 10.1038/ncomms11457 (PMC4855534; doi:10.1038/ncomms11457)
Supplement: Supplementary Information — Supplementary Figures 1-6 [file ncomms11457-s1.pdf]

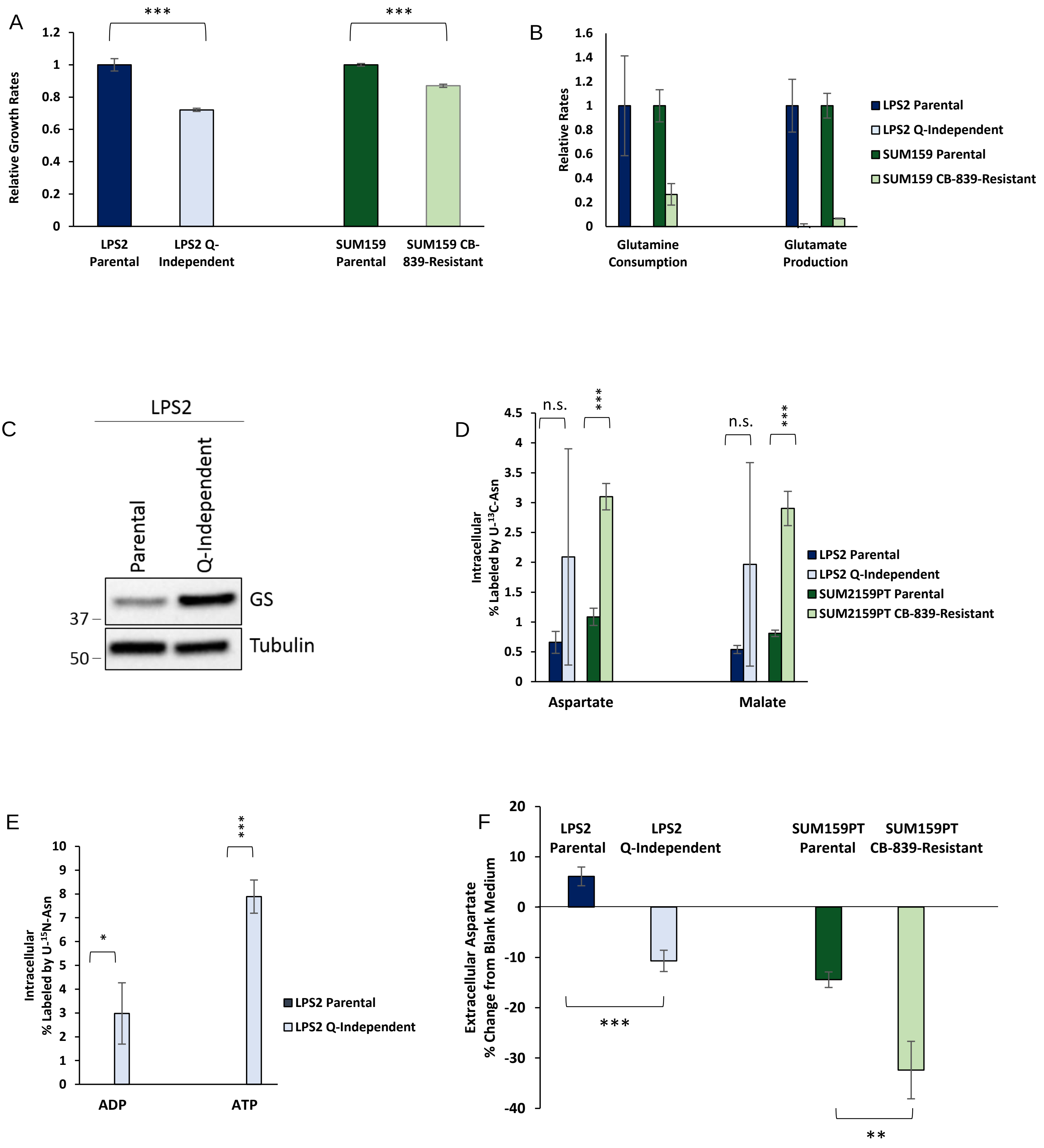
A
***
***
Relative Growth Rates
1.2
1
0.8
0.6
0.4
0.2
0
LPS2 Parental
LPS2 Q-Independent
SUM159 Parental
SUM159 CB-839-Resistant
B
Relative Rates
1.6
1.4
1.2
1
0.8
0.6
0.4
0.2
0
Glutamine Consumption
Glutamate Production
LPS2 Parental
LPS2 Q-Independent
SUM159 Parental
SUM159 CB-839-Resistant
C
LPS2
Parental
Q-Independent
GS
Tubulin
37
50
D
Intracellular
% Labeled by U-13C-Asn
4.5
4
3.5
3
2.5
2
1.5
1
0.5
0
n.s.
***
n.s.
***
Aspartate
Malate
LPS2 Parental
LPS2 Q-Independent
SUM2159PT Parental
SUM2159PT CB-839-Resistant
E
Intracellular
% Labeled by U-15N-Asn
10
9
8
7
6
5
4
3
2
1
0
*
***
ADP
ATP
LPS2 Parental
LPS2 Q-Independent
F
Extracellular Aspartate
% Change from Blank Medium
20
10
0
-10
-20
-30
-40
LPS2 Parental
LPS2 Q-Independent
SUM159PT Parental
SUM159PT CB-839-Resistant
***
**

Supplementary Figure 1. Glutamine-independent and CB-839-resistant cells exhibit minimal asparagine metabolism. (a) Relative growth rates of LPS2 parental, LPS2 glutamine (Q)-independent, SUM159PT parental, and SUM159PT CB-839-resistant cells, normalized to the respective parental cell line. (**b**) Relative glutamine consumption and glutamate production rates for LPS2 parental, LPS2 glutamine (Q)-independent, SUM159PT parental, and SUM159PT CB-839-resistant cells, normalized to the respective parental cell line. (**c**) Immunoblot showing levels of glutamine synthetase (GS) and tubulin in parental and glutamine-independent LPS2 cells. (**d**) Percentages of intracellular ^{13}C-labeled aspartate and malate in LPS2 parental and glutamine-independent as well as SUM159PT parental and CB-839-resistant cells labeled with U-^{13}C-asparagine in the medium for 24 hours as determined by LC-MS. (**e**) Percentages of intracellular ^{15}N-labeled ADP and ATP in LPS2 parental and glutamine-independent cells labeled with U-^{15}N-asparagine in the medium for 24 hours as determined by LC-MS. (**f**) Changes in extracellular aspartate levels during a 24 hour incubation with the indicated cell line. Values are shown as percent change from aspartate measurements from identical medium incubated on plates lacking cells, with negative bars indicating cellular consumption and positive bars indicating production. Error bars denote standard deviation of the mean (n = 3). P values were calculated by the Student's t-test: $^*p < 0.05$; $^{**}p < 0.01$; $^{***}p < 0.001$; n.s., not significant.

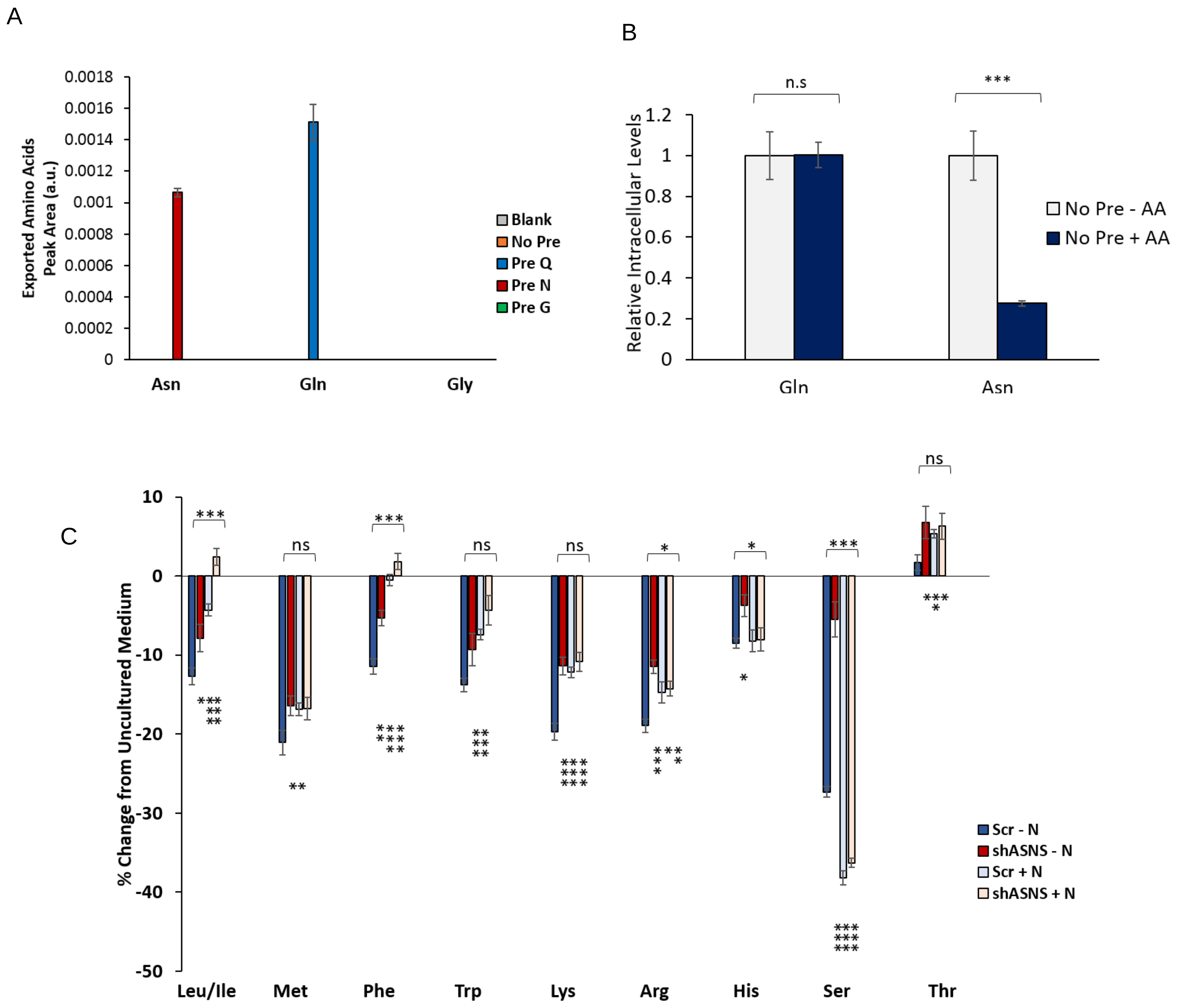

Supplementary Figure 2. Intracellular asparagine exchanges with extracellular amino acids to promote amino acid uptake. (**a**) Peak area for exported asparagine, glutamine, and glycine following amino acid stimulation of pre-loaded LPS2 cells. Glycine export was not detected. Cells were pre-loaded with 2 mM asparagine, glutamine, or glycine, respectively. (**b**) Relative intracellular glutamine and asparagine levels as measured by LC-MS in serum- and amino acid-starved LPS2 cells before (- AA) and after (+ AA) amino acid stimulation for 30 minutes. "No Pre" indicates lack of glutamine or asparagine pre-load. (**c**) Changes in extracellular amino acid levels during a 24 hour incubation with HeLa cells. The 24 hour incubation began 48 hours post-doxycycline-induced expression of a scrambled shRNA (Scr) or ASNS shRNA. Values are shown as percent change from amino acid measurements from identical medium incubated on plates lacking cells, with negative bars indicating cellular consumption and positive bars indicating production. Reduced import of certain amino acids upon asparagine supplementation may indicate competition with asparagine for a common transporter for import. For (a) and (b), error bars denote standard deviation of the mean (n = 3). For (c), error bars denote standard error of the mean (n = 6). P values were calculated by the Student's t-test: *p < 0.05; **p < 0.01; ***p < 0.001; ns, not significant.

A

	Gene	Breast	Lung	Pancreas	Colon	Head&Neck	Glioblastoma	Bladder	Thyroid	Sarcoma	Kidney	Melanoma	Liver
Serine Synthesis	PHGDH		0.33	0.66	0.83	0.43	0.31		0.53	0.40			
	PSAT1	0.72	0.50	0.63	0.83	0.76	0.36	0.75	0.85	0.58			
	PSPH		0.47			0.40							
	SHMT1												
	SHMT2	0.39	0.54	0.68		0.38		0.64	0.84				
Glycine/Folate Metabolism	MTHFD1L	0.54			0.58								0.46
	MTHFD2L						0.34						
	MTHFD1						0.39						-0.31
	MTHFD2	0.56	0.62	0.69	0.49	0.73	0.34	0.57	0.81		0.50	0.62	
	MTHFR						-0.39						

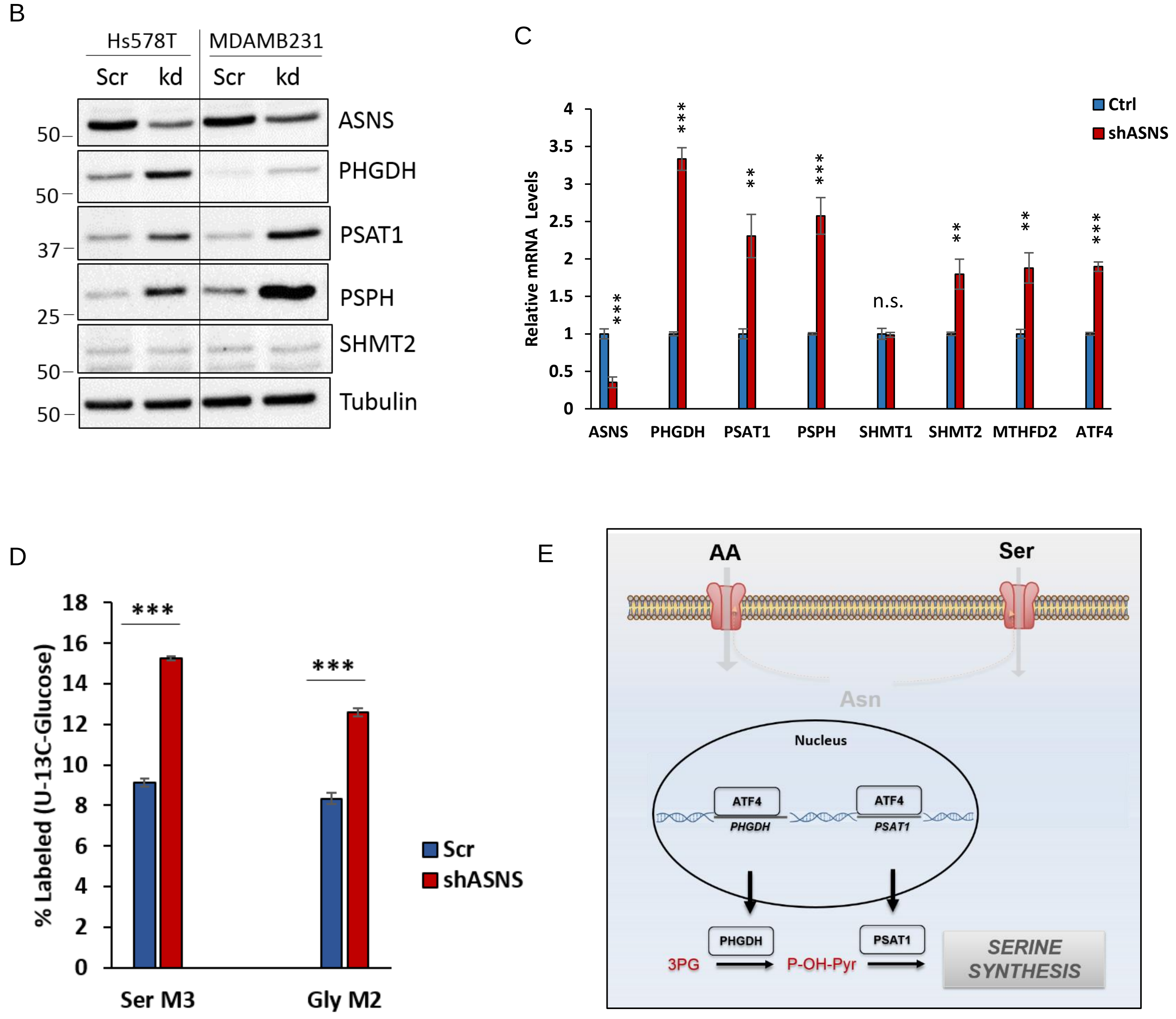

Supplementary Figure 3. Intracellular asparagine levels influence serine metabolism.
(**a**) Correlations between ASNS expression and expression of genes involved in serine/glycine metabolism in human tumors. Listed values are Pearson's correlation coefficients between ASNS mRNA levels and mRNA levels for the listed gene and for the indicated tumor. Blank cells indicate that expression is not substantially correlated with ASNS expression with a Pearson's correlation coefficient greater than 0.3 or less than -0.3. PHGDH, phosphoglycerate dehydrogenase; PSAT1, phosphoserine aminotransferase 1; SHMT1 and SHMT2, serine hydroxymethyltransferase 1 and 2; MTHFD1L, Methylenetetrahydrofolate dehydrogenase 1-like; MTHFD2L, Methylenetetrahydrofolate dehydrogenase 2-like; MTHFD1 and MTFHD2, methylenetetrathydrofolate dehydrogenase 1 and 2; MTHFR, methylenetetrahydrofolate reductase.
(**b**) Immunoblots comparing levels of proteins involved in serine/glycine biosynthesis in HS578T and MDAMB231 cells stably expressing scrambled shRNA (Scr) or ASNS shRNA (kd). Lysates were generated from cells cultured in DMEM (0 mM Asn) and were probed for ASNS, phosphoglycerate dehydrogenase (PHGDH), phosphoserine aminotransferase 1 (PSAT1), phosphoserine phosphatase (PSPH), serine hydroxymethyltransferase 2 (SHMT2), and tubulin. (**c**) Relative mRNA levels of genes involved in serine/glycine metabolism from HeLa cells 48 hours post-doxycycline induction of scrambled shRNA or ASNS shRNA. Quantitative real-time PCR was performed with primers specific to ASNS, PHGDH, PSAT1, PSPH, SHMT1, SHMT2, methylenetetrahydrofolate dehydrogenase 2 (MTHFD2), and activating transcription factor 4 (ATF4). (**d**) Percentages of intracellular ^{13}C-labeled serine and glycine in HeLa cells labeled with U-^{13}C-glucose for 24 hours at 24 hours post-induction of scrambled shRNA (Scr) or ASNS shRNA expression, as determined by LC-MS. Error bars denote standard deviation of the mean (n =3). P values were calculated by the Student's t-test: *p < 0.05; **p < 0.01; ***p < 0.001. (**e**) Schematic illustrating ATF4 activation of serine synthesis pathway gene expression and serine synthesis pathway flux in response to low intracellular asparagine levels.

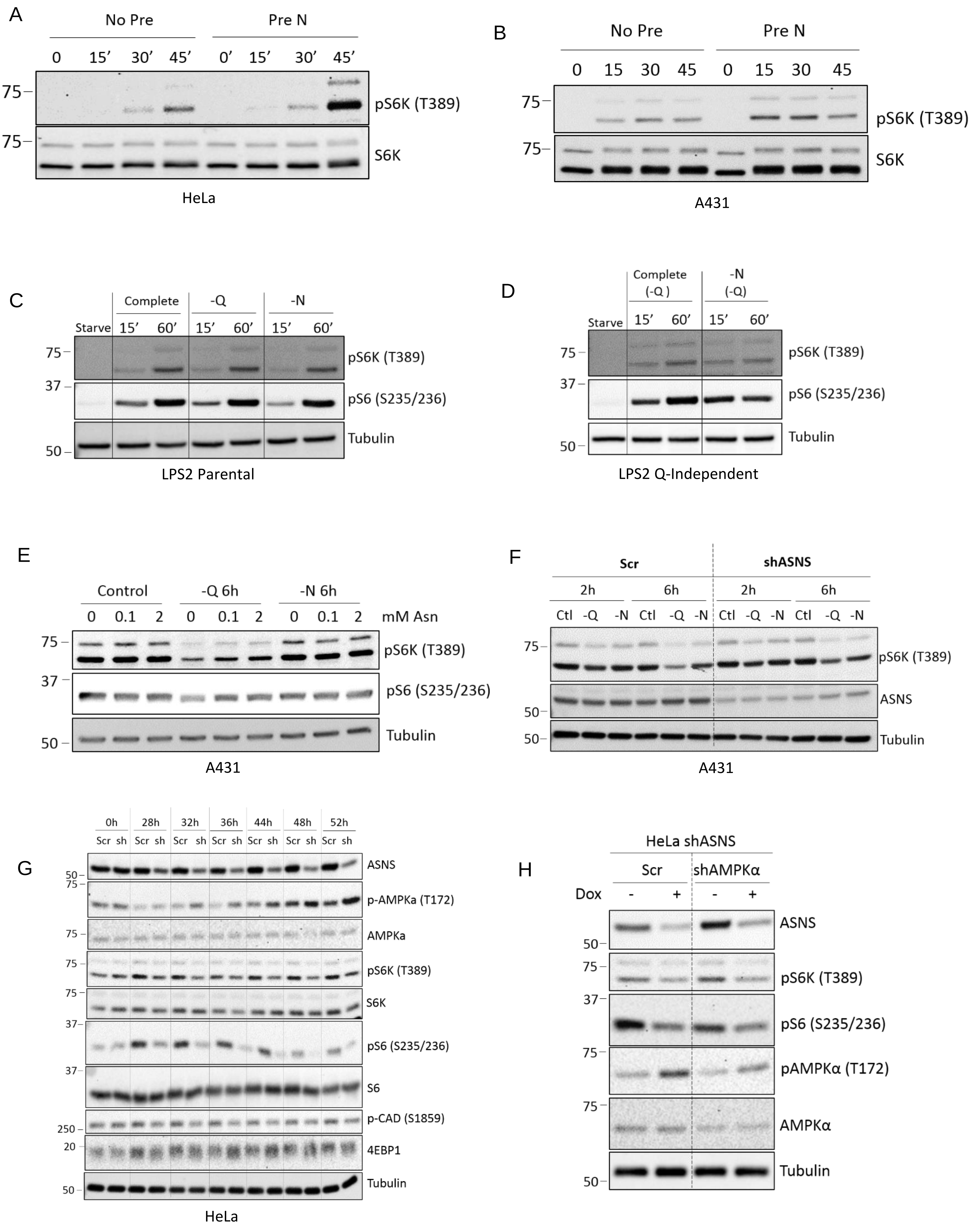
A
No Pre
Pre N
0 15’ 30’ 45’ 0’ 15’ 30’ 45’
75
pS6K (T389)
75
S6K
HeLa
B
No Pre
Pre N
0 15 30 45 0 15 30 45
75
pS6K (T389)
75
S6K
A431
C
Starve
Complete
-Q
-N
15’ 60’ 15’ 60’ 15’ 60’
75
pS6K (T389)
37
pS6 (S235/236)
50
Tubulin
LPS2 Parental
D
Starve
Complete (-Q)
-N (-Q)
15’ 60’ 15’ 60’
75
pS6K (T389)
37
pS6 (S235/236)
50
Tubulin
LPS2 Q-Independent
E
Control
-Q 6h
-N 6h
0 0.1 2 0 0.1 2 0 0.1 2 mM Asn
75
pS6K (T389)
37
pS6 (S235/236)
50
Tubulin
A431
F
Scr
shASNS
2h 6h 2h 6h
Ctl -Q -N Ctl -Q -N Ctl -Q -N Ctl -Q -N
75
pS6K (T389)
50
ASNS
50
Tubulin
A431
G
0h 28h 32h 36h 44h 48h 52h
Scr sh Scr sh Scr sh Scr sh Scr sh Scr sh Scr sh
50
ASNS
75
p-AMPKa (T172)
75
AMPKa
75
pS6K (T389)
75
S6K
37
pS6 (S235/236)
37
S6
250
p-CAD (S1859)
20
4EBP1
50
Tubulin
HeLa
H
HeLa shASNS
Scr
shAMPKα
Dox - + - +
50
ASNS
75
pS6K (T389)
37
pS6 (S235/236)
75
pAMPKα (T172)
75
AMPKα
50
Tubulin

Supplementary Figure 4. Intracellular asparagine levels regulate mTORC1 activation.
(**a,b**) Immunoblotting of lysates from starved HeLa and A431 cells pre-loaded with starve medium (No Pre) or asparagine (Pre N) following amino acid stimulation for the indicated times. Lysates were probed for phosphorylation of S6K at T389 and total S6K. (**c**) Immunoblot showing phosphorylation of S6K (T389) and S6 ribosomal protein (S235/236) following stimulation of starved LPS2 parental cells with amino acids for the indicated times. Starved cells were stimulated with serum-free m-DMEM (Complete), serum-free m-DMEM lacking glutamine (-Q), serum-free m-DMEM lacking asparagine (-N), or left unstimulated (Starve). (**d**) Immunoblot showing phosphorylation of S6K phosphorylation (T389) and S6 ribosomal protein (S235/236) following stimulation of starved LPS2 glutamine-independent cells with amino acids for the indicated times. Starved cells were stimulated with serum- and glutamine-free m-DMEM (Complete), serum- and glutamine-free m-DMEM lacking asparagine (-N), serum-free m-DMEM lacking asparagine (-N), or left unstimulated (Starve). (**e**) Immunoblot showing S6K phosphorylation (T389) following starvation of A431 cells of glutamine (-Q) or asparagine (-N) for 6 hours. Prior to starvation, cells were cultured for 7 days in DMEM supplemented with the indicated concentration of asparagine.
(**f**) Immunoblot showing phosphorylation of S6K (T389) in A431 cells stably expressing scrambled shRNA or ASNS shRNA with and without (Ctl) starvation of glutamine (-Q) or asparagine (-N) for the indicated times. (**g**) Immunoblot showing ASNS, S6K phosphorylation (T389), S6 phosphorylation (S235/236), AMPKα phosphorylation (T172), and total S6K, S6, and AMPKα in HeLa cells at the indicated times post-doxycycline induction of scrambled shRNA (Scr) or ASNS shRNA expression. (**h**) Immunoblot showing ASNS, S6K phosphorylation (T389), S6 phosphorylation (S235/236), AMPKα phosphorylation (T172), and total AMPKα protein levels with or without a 48 hour doxycycline induction of ASNS shRNA in HeLa cells stably expressing scrambled or AMPKα shRNA.

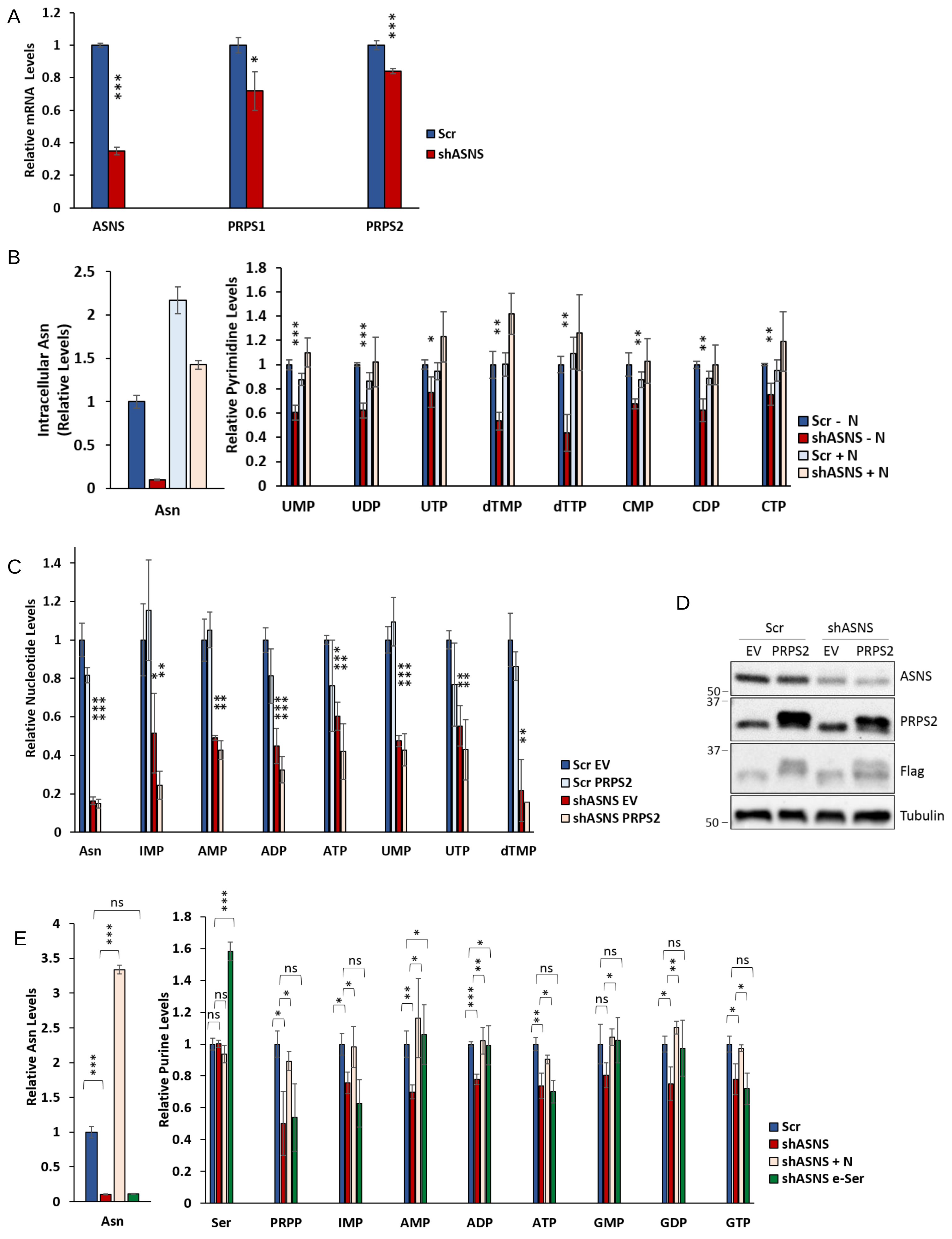
A
Relative mRNA Levels
ASNS
PRPS1
PRPS2
Scr
shASNS
B
Intracellular Asn (Relative Levels)
Asn
Relative Pyrimidine Levels
UMP
UDP
UTP
dTMP
dTTP
CMP
CDP
CTP
Scr - N
shASNS - N
Scr + N
shASNS + N
C
Relative Nucleotide Levels
Asn
IMP
AMP
ADP
ATP
UMP
UTP
dTMP
Scr EV
Scr PRPS2
shASNS EV
shASNS PRPS2
D
Scr
shASNS
EV
PRPS2
EV
PRPS2
ASNS
PRPS2
Flag
Tubulin
50
37
37
50
E
Relative Asn Levels
Asn
Relative Purine Levels
Ser
PRPP
IMP
AMP
ADP
ATP
GMP
GDP
GTP
Scr
shASNS
shASNS + N
shASNS e-Ser

Supplementary Figure 5. Intracellular asparagine levels regulate purine and pyrimidine synthesis. (**a**) Relative PRPS1 and PRPS2 mRNA levels from HeLa cells 48 hours post-doxycycline induction of scrambled shRNA or ASNS shRNA. Quantitative real-time PCR was performed with primers specific to PRPS1 and PRPS2. (**b**) Relative levels of the indicated intracellular metabolites extracted from HeLa cells 48 hours after doxycycline induction of scrambled shRNA (Scr) or ASNS shRNA expression as measured by LC-MS. (**c**) Relative levels of the indicated intracellular metabolites extracted from HeLa cells stably expressing PRPS2 or possessing empty pLHCX vector (EV) 72 hours after doxycycline induction of scrambled shRNA (Scr) or ASNS shRNA expression as measured by LC-MS. (**d**) Immunoblot confirming PRPS2 expression in HeLa cells indicated in (c). (**e**) Relative levels of the indicated intracellular metabolites extracted from HeLa cells 48 hours post-doxycycline induction of scrambled shRNA or ASNS shRNA in the presence of 0.1 mM asparagine (+ N), 0.2 mM serine methyl-ester (+ e-Ser), or unsupplemented DMEM..Error bars denote standard deviation of the mean (n = 3). P values were calculated by the Student's t-test: *$p < 0.05$; **$p < 0.01$; n.s., not significant.

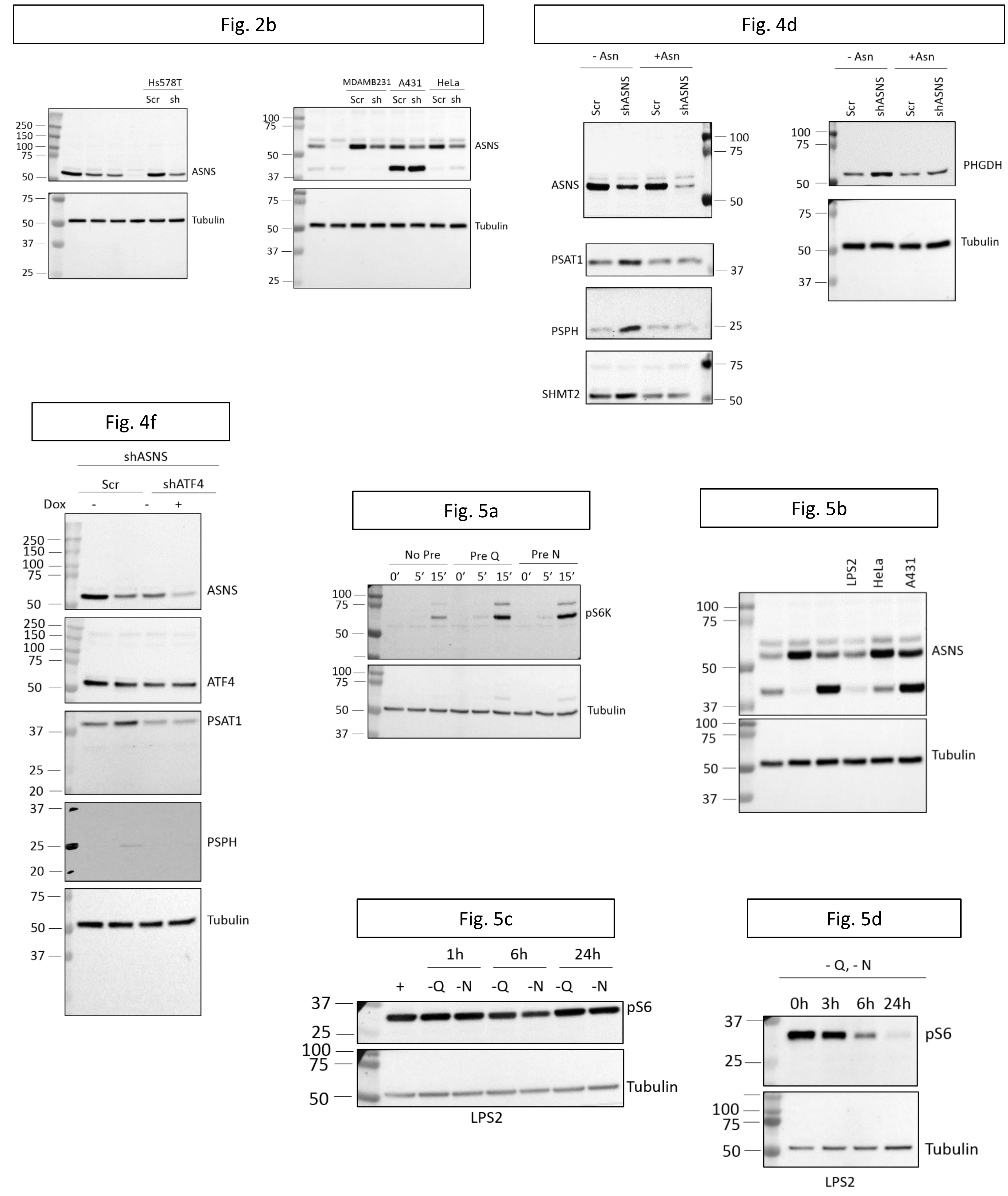

Supplementary Figure 6. Uncropped western blot images presented in this manuscript. Labeling above each image indicates the corresponding figure in the main manuscript.

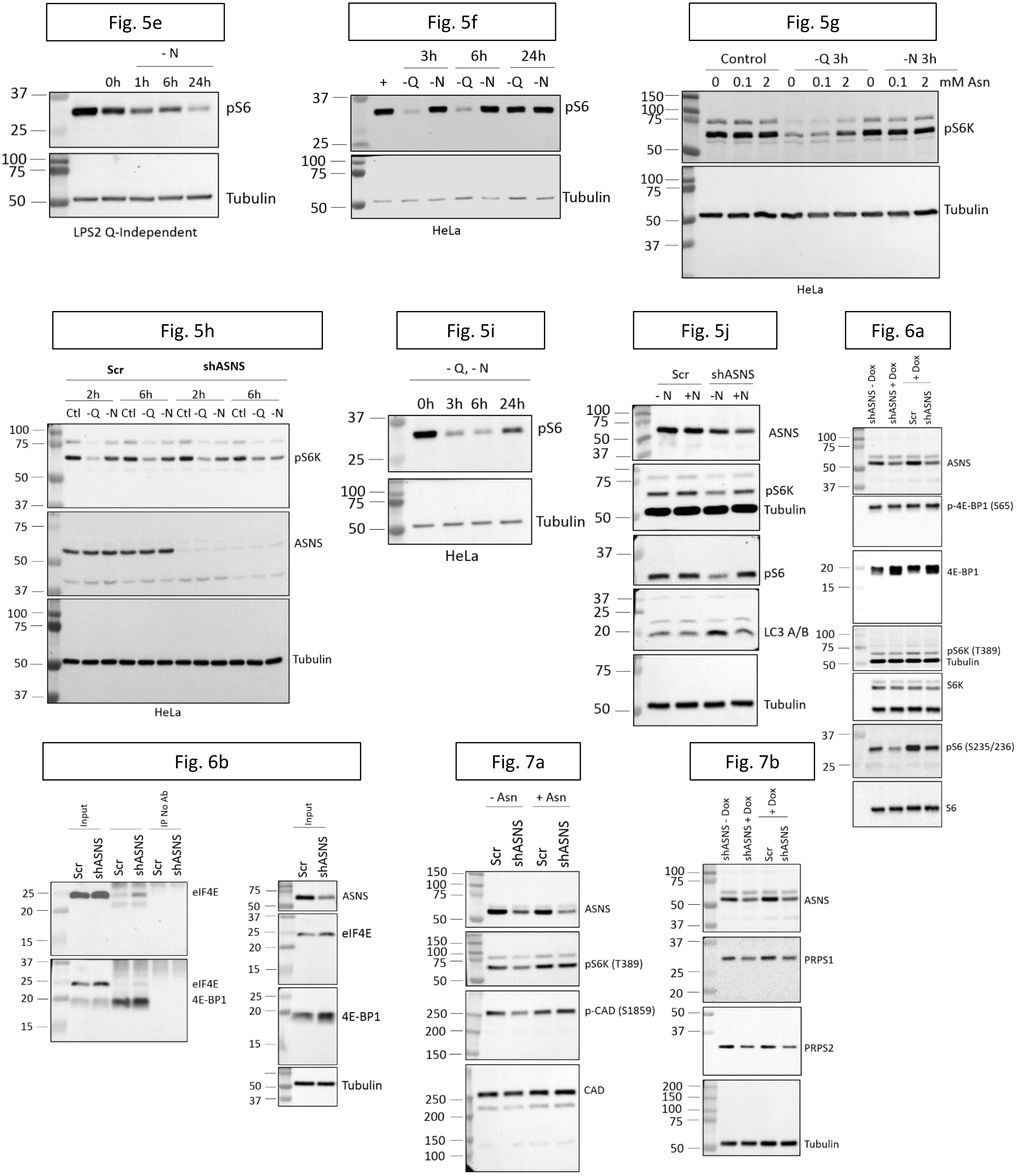

Supplementary Figure 6. Uncropped western blot images presented in this manuscript. Labeling above each image indicates the corresponding figure in the main manuscript.
